# Forensic interventions carried out by nurses on older people in situations of violence: a comparative study*

**DOI:** 10.1590/1980-220X-REEUSP-2024-0282en

**Published:** 2025-05-09

**Authors:** Jiovana de Souza Santos, Francisca de Chagas Alves de Almeida, Jefferson da Silva Soares, Isabel Vitória do Nascimento Pereira Gomes, Raúl Fernando Guerrero Castañeda, Rafaella Queiroga Souto

**Affiliations:** 1 Universidade Federal do Pernambuco, Centro de Ciências da Saúde, Departamento de Enfermagem, Recife, PE, Brazil.; 2 Hospital Universitário Lauro Wanderley, João Pessoa, PB, Brazill.; 3 Universidade Federal da Paraíba, Centro de Ciências da Saúde, Curso de Enfermagem, João Pessoa, PB, Brazil.; 4 Universidade Federal da Paraíba, Centro de Ciências da Saúde, Curso de Graduação em Enfermagem, João Pessoa, PB, Brazil.; 5 Universidad de Guanajuato, Guanajuato, Gto, México.; 6 Universidade Federal da Paraíba, Centro de Ciências da Saúde, Departamento de Saúde Coletiva, João Pessoa, PB, Brazil.

**Keywords:** Nursing Care, Forensic Nursing, Elder Abuse, Violence, Nurses Improving Care for Health System Elders

## Abstract

**Objective:**

To investigate forensic interventions carried out by nurses with and without experience in caring for older people in situations of violence.

**Method:**

Cross-sectional, quantitative study with nurses working in five hospitals in João Pessoa, Paraíba, Brazil. Data were collected with a structured questionnaire, validated by ten experts and with good reliability, presenting a Cronbach's alpha coefficient greater than 0.70. Data were analyzed using simple and relative frequency, Bayesian test for proportions, Student’s t test, multivariate analysis with multiple correspondence and cluster analysis models with Chi-Square metric with hierarchical clustering form and average linkage method.

**Results:**

A total of 270 nurses participated, of which 203 had no experience with older people in situations of violence. Within the groups, a significant difference was observed (P < 0.001), that is, the interventions of the group with experience are more consistent with ethical-legal aspects than those without experience.

**Conclusion:**

Significant similarity was found between the two groups, in which experienced nurses carried out appropriate and relevant interventions, although the execution of actions to interrupt the cycle of violence presented weaknesses.

## INTRODUCTION

Violence has hospitalization as a significant consequence and, in this context, health professionals play a relevant role in identifying cases, since they are directly assisting users and have the opportunity, sometimes unique, to identify the situation. However, due to the weaknesses presented by nurses in their skills to care for older people in situations of violence, effective responses have not been achieved^(1)^. Based on this premise, in this decade, forensic nursing (FN) has been expanding in Brazil, enabling contribution and performance in the work process so that nurses can develop a holistic vision to identify cases of violence^(2)^, through the combination of nursing science, forensic science, and specific health care.

Forensic nursing is a specialized area that combines nursing practice with clinical and legal knowledge, serving both living or deceased victims, and offenders. Its main responsibilities include detecting signs of violence through history taking and physical examination, collecting, preserving and storing evidence, and collaborating with the judicial system through complaints and statements. Furthermore, it plays a fundamental role in providing comprehensive and humanized assistance to victims of violence^(3)^.

In this scenario, it is up to the nurse to identify forensic injuries, document, guide, report, collect, and preserve evidence. Moreover, the principle of confidentiality must be followed and the data from the collection of all information about the evidence of the crime preserved. Forensic patients, in addition to physical and psychological care, also need to have their legal rights protected. To ensure these rights, evidence registration and collection, as well as preservation of the chain of custody, are essential^(4)^.

The literature highlights that any evidence obtained increases the rates of prosecution and conviction for crimes, which results in perpetrators receiving longer sentences and, most importantly, that this leads to the rupture of the cycle of violence^(5)^. From this perspective, the Federal Nursing Council (COFEN) highlights that nurses have an understanding of the health, social, and legal systems, enriched by knowledge of forensic sciences and public health, and can work with the criminal system, government agents, and interpretations of forensic injuries^(6)^.

The concept of forensic injury can be defined as damage caused to the physical integrity of a person, consisting of any change in the structure or function of the human body resulting from external factors, the analysis of which is carried out for the purposes of judicial investigation, identifying the cause, the extent of the damage, and the instrument used^(7)^.

In this context, forensic intervention is a process that involves the application of technical and scientific knowledge in investigations that require clarifications of a legal nature, with the aim of supporting Justice. Thus, it seeks to provide technical elements for decision-making by judicial authorities^(7)^. This is an interdisciplinary intervention and, with regard to nursing, examples include warm embracement, active listening, recording of physical injuries, collection of biological traces, such as semen, and preservation of the integrity of evidence^(8)^.

The role of nurses in dealing with violence encompasses all genders and age groups; however, this study emphasizes violence against older people (VAOP), which is considered a public health problem that requires effective interventions. In Brazil, a multicenter study carried out in two hospitals, with 323 older people, showed that more than half of the participants were at risk of violence^(9)^.

Therefore, revealing the potential and weaknesses of nursing performance regarding the nuances involved in managing VAOP is required, as this shows the reality and allows interventions. Therefore, preparing this professional to handle cases of violence means considering that their work involves careful observation of the older person’s daily life, giving them the opportunity to prevent, detect, and interrupt the action^(10)^.

In view of the above, the following questions arise: how does or will the nurse intervene in cases of VAOP in the hospital context? Are there differences in interventions among nurses who attended and did not attend VAOP cases? To answer these questions, we investigated forensic interventions carried out by nurses with and without experience in caring for older people in situations of violence. Thus, this study is justified as it considers that research on nurses’ interventions in VAOP in hospitals is still scarce and highlighting the weaknesses and potentialities of this action is essential for decision-making.

## METHOD

### Design of Study

This is a descriptive cross-sectional study, with a quantitative approach, conducted based on the recommendations of the tool *STrengthening the Reporting of OBservational studies in Epidemiology* (STROBE).

### Study Local

The study was carried out in five public hospitals in João Pessoa, PB, Brazil, selected for serving a significant number of older people, in the following sectors: emergency, internal medicine, surgical clinic, and Intensive Care Unit (ICU).

### Population and Selection Criteria

The population consisted of up of 660 nurses, of which 285 were distributed across the four sectors selected. The eligibility criteria were: being nurses with at least four weeks of experience in the sector and 20 hours of work per week. This minimum period was considered because it is necessary for the professional to be exposed to practical situations inherent to his/her profession, which allows him/her to respond effectively to the research^(11)^. Nurses who performed exclusively managerial activities and those who were away from work for any reason were excluded.

### Sample Definition

Considering the accessible population as being all nurses from the four sectors, we have a finite population of size N = 285. Thus, a simple random sample with 98% confidence and a 2% margin of error was considered. The sample size was obtained by applying the formula below, where z = 1.96; P = 0.50; Q = 1−P; N = 285 and d = 0.03, and with finite population correction with the expression n_f_ ≈ n / [1+ n /N]^(12)^. Thus, 253 were required for minimum sampling, but 270 were collected.

$$n=\frac{\frac{z^{2}PQ}{d^{2}}}{1+\frac{1}{N}\left(\frac{z^{2}PQ}{d^{2}}-1\right)}$$

### Data Collection

The collection took place from June to December 2022, through a structured questionnaire developed by the researcher and validated by 10 experts. To apply the instrument to participants, a convenience approach was taken, meeting professionals at available times. The researcher made weekly visits to the institutions’ sectors, inviting active nurses to participate. The questionnaire was applied at the location indicated by the participants themselves, strictly following the protocol for wearing surgical masks, in accordance with Covid-19 prevention measures.

To prepare the questionnaire, the scoping review “Forensic nursing care for older people in situations of violence: a scoping review” was carried out to identify how this care takes place. The instrument items showed good reliability with Cronbach’s Alpha greater than 0.70, considered a good measure^(13)^, showing that the instrument allows identifying nurses’ interventions in the face of VAOP. Furthermore, the point-biserial correlation coefficient between each item presents values below 0.50, showing that all items in the instrument are important. The probability of the participant having “guessed” an answer, guessing at random, was less than 0.04, with up to 0.05 being acceptable^(14)^.

The questionnaire consisted of sociodemographic data and general aspects of VAOP, covering 11 items, identified by the letter “A” followed by an Arabic number of the item; and actions performed by nurses in cases of VAOP, with 16 items, identified by the letter “B” and a sequence of Arabic numbers with answers “yes”, “no” and “maybe”. To apply the instrument, a convenience approach was used, with professionals being met, taking their availability into account.

### Data Analysis and Treatment

Data were organized in an Excel (Microsoft 365, 2019) spreadsheet and the analysis performed with the free software Jamovi Version 0.9, 2020 and SPSS (*Statistical Package for the Social Sciences*) version 20.0. To characterize sociodemographic data and VAOP, simple and relative frequency were used. Regarding the distinction between groups with and without experience, the proportion test was applied. If it was impossible to apply this test (proportions close to 0 or 1), comparison was used through the Bayesian test for proportions. For association between quantitative variables, the Student’s t test was performed and for differentiation of items with the highest and lowest percentages of responses, multivariate statistical analysis was used, applying Multiple Correspondence Analysis (MCA) with presentation of the results on a perceptual map and cluster analysis with the Chi-Square metric, with hierarchical clustering and average linkage method in dendrogram^(14,15)^. The internal consistency of the instrument was analyzed with the *Cronbach’s alpha*, with a 95% confidence interval.

### Ethical Aspects

This research is part of the project Instrumentalization of forensic nursing in the care of hospitalized older people, approved by the Research Ethics Committee with Opinion no. 3.709.600 and CAAE no. 10179719.9.0000.5183, following Resolution 466/2012 of the National Health Council.

## RESULTS

A total of 270 nurses participated in the research. Of these, 203 (75%) had no experience with VAOP and 67 (25%) had. It is observed, in both groups - with and without experience in VAOP, that the majority of participants are women (236 − 87.4%), in the age range of 31 to 40 years (172 − 63.8%), specialists (244 − 90.4%), working in the emergency (103 − 38.1%) and internal medicine (69 − 25.6%) departments with at least two jobs (204 − 75.5%). Regarding time of experience as a nurse, participants without experience in VAOP had more than ten years of experience in the field (60 – 29.5%), while among participants with experience in VAOP 30 (44.7%) had three to six years of experience in the field, as shown in Table 1.

**Table 1:** Sociodemographic variables of nurses – João Pessoa, PB, Brazil, 2022.

Variable	Category	Without		With		Total	
		N	%	N	%	N	%
**Sex**	Male	24	8.8	10	3.7	34	12.6
	Female	179	66.2	57	21.1	236	87.4
**Age**	18 a 30	4	1.5	14	5.1	18	6.7
	31 to 40	131	48.5	41	15.1	172	63.8
	41 to 50	67	24.8	8	2.9	75	27.8
	60 to 70	1	0.4	4	1.5	5	1.9
**Level of education**	PhD	2	0.7	1	0.3	3	1.1
	Master’s degree	5	1.8	4	1.5	9	3.3
	Specialist	188	69.6	56	20.7	244	90.4
	Undergraduate	8	2.9	6	2.2	14	5.2
**Sector**	Surgical Clinic	38	14.0	10	3.7	48	17.8
	Internal Medicine	40	14.8	29	10.7	69	25.6
	Urgency	89	32.9	14	5.1	103	38.1
	ICU	36	13.3	14	5.1	50	18.5
**Number of jobs**	One	7	2.6	21	7.8	28	10.4
	Two	165	61.1	39	14.4	204	75.5
	Three	30	11.1	7	2.59	37	13.7
	Four	1	0.3	0	0.0	1	0.3
**Time working in the sector (in years)**	≤1	10	4.9	5	7.4	15	5.6
	1 to 3	55	27.0	15	22.3	70	25.9
	3 to 6	38	18.7	30	44.7	68	25.2
	6 to 9	40	19.7	7	10.4	47	17.4
	=10	60	29.5	10	14.9	70	25.9

Regarding the general aspects of VAOP, only in item A5 was there a similarity in the responses between the two groups, and a significant difference in < 0.005 in the other items as shown in Table 2.

**Table 2:** General aspects about VAOP considering the responses of the two groups – João Pessoa, PB, Brazil, 2022.

Item	Category	Without		With		Total		p value Fisher’s test
		N	%	N	%	N	%	
A1 – Have you identified any case or suspicion of Violence against older people?	**Yes**	0	0.0	67	100	67	24.8	
	**No**	198	97.5	0	0.0	198	73.3	< 0.001*
	**Maybe**	5	2.5	0	0.0	5	1.9	
A2 – Have you ever noticed any discomfort in the older people in the presence of their family members?	**Yes**	74	36.5	62	92.5	136	50.4	
	**No**	128	63.1	4	6.0	132	48.9	< 0.001*
	**Maybe**	1	0.5	1	1.5	2	0.7	
A3 – Do you find it easy to detect that an older person is in a situation of violence?	**Yes**	35	17.2	19	28.4	54	20.0	
	**No**	165	81.3	42	62.7	207	76.7	0.0014*
	**Maybe**	3	1.5	6	9.0	9	3.3	
A4 – Do you know of any instrument or scale that helps in providing assistance to older people with suspected or confirmed violence? (even if you have never used it)	**Yes**	2	1.0	9	13.4	11	4.1	
	**No**	201	99.0	56	83.6	257	95.2	
	**Maybe**	0	0.0	2	3.0	2	0.7	< 0.001*
A5 – Do you consider it important to have some material (protocol, scale, instrument) to help in the care of older people who are victims of violence?	**Yes**	201	99.0	66	68.5	267	98.9	
	**No**	2	1.0	1	1.5	3	1.1	0.9999*
	**Maybe**	0	0.0	0	0.0	0	0.0	
A6 – Do you use any instrument or scale that helps in providing assistance to older people with suspected or confirmed abuse? (even if you have never used it)	**Yes**	1	0.8	5	7.5	6	2.2	
	**No**	201	99.5	60	89.5	262	97.0	
	**Maybe**	0	0.0	2	3.0	2	0.7	0.0004*
A7 – Do you feel prepared to identify situations of violence against the older people?	**Yes**	63	31	11	16.4	74	27.4	
	**No**	133	65.5	47	70.1	180	66.7	0.0026*
	**Maybe**	7	3.5	9	13.4	16	5.9	
A8 – Do you think there is sufficient training available for nurses to identify situations of violence against older people?	**Yes**	0	0.0	7	10.4	7	2.6	
	**No**	203	100	59	88.1	262	97.0	
	**Maybe**	0	0.0	1	1.5	1	0.4	< 0.001*
A9 – Do you think that older people hide the fact that they have suffered violence?	**Yes**	146	71.9	62	92.5	234	86.7	
	**No**	57	77.3	3	4.5	33	12.3	< 0.001*
	**Maybe**	0	0.0	2	3.0	3	1.1	
A10 – Have you ever witnessed situations in which older people report being better cared for in the hospital than in their home environment?	**Yes**	146	71.9	62	92.5	108	40	
	**No**	57	28.0	3	4.5	160	59.3	
	**Maybe**	0	0.0	2	3.0	2	0.7	< 0.001*
A11 – Do you think that violence hinders the improvement of older people when they are hospitalized?	**Yes**	172	84.7	62	92.5	234	86.7	
	**No**	31	15.3	3	4.5	3	1.1	
	**Maybe**	0	0.0	2	3.0	33	12.2	0.0033*

*Fisher’s test. A = item categorization.

In table 3, it can be seen that there is a significant difference in most items in both groups, except for items B1 and B5. At the end of table 3, the Student’s t test*,* suitable for large samples when comparing averages, confirms that there is a significant difference between the scores obtained by the two groups.

**Table 3:** Comparison of nurses’ interventions with and without experience in VAOP – João Pessoa, PB, Brazil, 2022.

Item	Category	Without		With		Total		p value Chi-square
		N	%	N	%	N	%	
B1 – Did you refer or would you refer suspected or confirmed cases to the Social Worker?	**Yes** **No** **Maybe**	203 0 0	100 0 0	65 2 0	97.0 2.9 0	268 2 0	98.9 0.7 0.0	0.3075
B2 – Did you refer or would you refer suspected or confirmed cases to the police authorities?	**Yes** **No** **Maybe**	11 189 3	5.4 93.1 1.5	26 38 3	38.8 56.7 4.4	37 225 8	13.7 83.6 2.9	< 0.001*
B3 – Did you refer would you refer suspected or confirmed cases to the police authorities?	**Yes** **No** **Maybe**	13 189 1	6.4 93.1 0.5	26 40 4	38.8 59.7 6	39 224 7	14.4 83.3 2.6	< 0.001*
B4 – Do you perform anamnesis/interview on the older people to assess signs and symptoms of violence?	**Yes** **No** **Maybe**	201 1 0	99.0 0.5 0.0	59 7 1	88.1 10.4 1.5	260 7 3	95.9 2.6 2.1	< 0.001*
B5 – Do you carry out qualified listening to try to identify suspected cases of violence against older people?	**Yes** **No** **Maybe**	171 30 1	83.3 14.7 0.5	60 4 3	89.6 5.9 4.4	229 35 6	84.8 12.9 2.2	0.3429
B6 – Do you perform physical examination on the older people to assess signs and symptoms of violence?	**Yes** **No** **Maybe**	112 46 30	54.9 22.6 14.7	61 4 2	91.0 5.9 3	173 50 32	64.0 18.5 11.8	< 0.001*
B7 – Do you or would you record suspected or confirmed cases in the medical records?	**Yes** **No** **Maybe**	201 2 0	98.5 0.9 0.0	58 4 5	86.6 5.9 7.4	259 5 6	95.9 1.8 2.2	< 0.001*
B8 – Do you or would develop care plans for older people and families involved in situations of mistreatment, sexual abuse, trauma, and other forms of violence?	**Yes** **No** **Maybe**	42 160 1	20.6 78.8 0.5	27 39 1	40.3 58.2 1.5	69 199 2	25.5 73.7 0.7	0.0023*
B9 – Do you embrace older people who are victims of sexual violence, trauma, and other forms of violence?	**Yes** **No** **Maybe**	203 0 0	100 0.0 0.0	63 3 1	94.0 4.5 1.5	266 4 0	98.5 1.5 0.0	0.0178*
B10 – Do you question the older person’s family in case of suspicion or confirmation?	**Yes** **No** **Maybe**	103 52 40	50.7 25.6 19.7	57 5 5	85.1 7.4 7.4	160 57 45	59.2 21.1 16.6	< 0.001*
B11 – Do you report suspected or confirmed cases of violence against older people to the multidisciplinary team to try to solve the problem?	**Yes** **No** **Maybe**	203 0 0	100 0.0 0.0	61 5 1	91.0 7.4 1.5	265 4 1	98.1 1.4 0.3	0.667B**
B12 – In suspected or confirmed cases of violence against older people, inform the doctor of the situation so he/she can help you with the management.	**Yes** **No** **Maybe**	203 0 0	100 0.0 0.0	62 4 1	92.5 1.4 1.5	265 4 1	98.1 1.4 0.3	0.130B**
B13 – Collaborates with the judicial system when necessary.	**Yes** **No** **Maybe**	171 30 2	83.8 41.2 0.9	59 5 3	88.1 7.4 4.4	230 34 6	85.1 12.6 2.2	0.0039*
B14 – Recognizes possible situations of violence against older people, identifies potential victims and prepares nursing diagnoses in the context of mistreatment, trauma, sexual violence and other forms of violence.	**Yes** **No** **Maybe**	10 192 1	4.9 94.5 0.5	36 27 4	53.7 40 6	46 216 8	17.0 80 2.9	< 0.001*
B15 – Promotes or would promote the protection of human rights and legal guarantees of older people, their families, and the people who committed the violence.	**Yes** **No** **Maybe**	196 2 5	96.1 0.9 2.4	59 3 5	88.1 4.5 7.4	255 6 9	94.4 2.2 3.3	0.0340*
B16 – Preserves or would preserve traces in cases of mistreatment, sexual violence and other forms of violence for the purpose of proving violence against older people.	**Yes** **No** **Maybe**	13 187 3	6.4 92.1 1.4	27 38 2	40.2 56.7 2.9	54 208 8	20 77.0 2.9	< 0.001*
**Mean**		10.0		12.2		10.6		**p value**
**Median**		11.0		12.0		11.0		0.0020*t
**Standard deviation**		1.7		3.0		2.3		

*Significant difference at 5% level - Chi-square test.

**B = Used Bayesian two-proportion test.

*t = Student’s t-test. B = item categorization.

It can be seen that there are items with high response percentage values and others with very low ones in table 3, and to decide which items are differentiated with any of these characterizations, multivariate statistical analysis was used, applying the ACM models^(15)^ and cluster analysis^(16)^.

The ACM results presented in Table 4 allow us to conclude that a single dimension of items in Table 3 accounts for 98.4% of the total variability contained in the information in Table 3. Therefore, dimension 2 (items: B1, B4, B5, B7, B9, B11, B12, B15) presents items more associated with affirmative responses.

**Table 4:** Multiple Correspondence Analysis of the responses in Table 3 – João Pessoa, PB, Brazil, 2022.

Dimension	Singular value	Inertia	Chi-square*	p value	% Inertia
1	0.096	0.009	2379.93	< 0.001	1.6
2	0.740	0.548			98.4
Total		0.557			100.0

**Figure 1:**
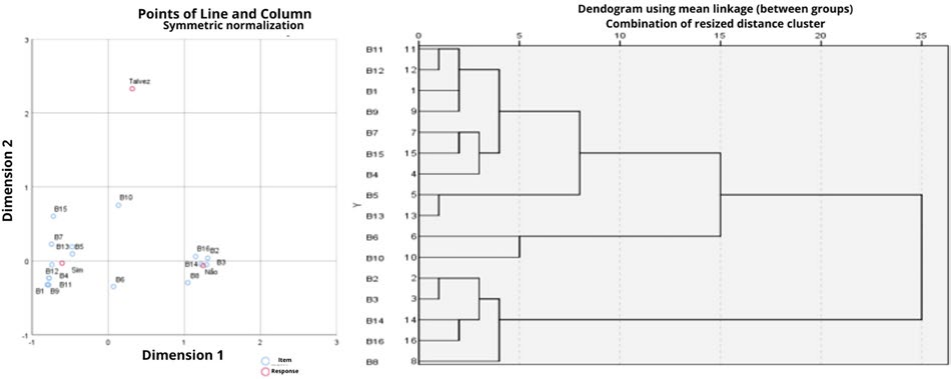
Perceptual map of the ACM and Dendrogram of the Cluster Analysis of Table 3 – João Pessoa, PB, Brazil, 2022.

In Figure 1, we have the perceptual map in two dimensions, where we can see that there are three clusters whose meaning, in the ACM, is that the greater the proximity between the levels of the categories in Table 3, the greater the association between these elements forming the cluster^(16)^. The largest cluster is formed by the association between items B1, B4, B5, B7, B9, B11, B12, B13 and B15 and the answer “yes”.

It can be seen that the common characteristic of all these items is their high percentage of occurrence. Items B6 and B10 are more associated with “maybe” responses for the group without experience in VAOP and, finally, the cluster on the right formed by items B2, B3, B8, B14 and B16, is the one that presents items with the lowest percentages of “yes” responses, being more associated with “no” responses for both groups.

Cluster analysis with Chi-Square metric with hierarchical clustering form and mean linkage method^(15)^ has its dendrogram presented next to the perceptual map, and a vertical cut is observed at a distance equal to 10, where the same groups of items presented in the ACM are observed, confirming the same interpretation given to the results presented in the perceptual map.

## DISCUSSION

It was revealed that, regarding the general aspects of VAOP, most nurses, in both groups, have noticed discomfort in the older person in the presence of a family member, found it difficult to detect VAOP, reported not knowing and not using instruments and/or protocols that help in the identification and intervention of cases, and found it important to have them. Furthermore, nurses do not feel prepared to identify VAOP, reporting that there is not enough training available to prepare them, which makes it difficult to manage cases, since the older person hides the violence.

In line with the findings, studies on the perceptions of nursing professionals regarding the detection and prevention of VAOP reveal that, although many consider or suspect the existence of cases, they face difficulties in acting, especially due to the lack of specific training and the absence of integration between public services aimed at this type of care. Moreover, they show concern about the lack of preparation^(17,18)^.

Given the lack of preparation revealed in this study and in other research, it is important to emphasize that nurses play a significant role in the legal implications of forensic cases and, to this end, they need to acquire knowledge of nursing in forensic sciences^(19)^. Furthermore, the use of protocols and instruments that guide the conduct of VAOP are fundamental for adequate assistance^(20)^.

In this research, it was noted that the majority of nurses revealed that they had already witnessed older people reporting that they were better cared for in the hospital environment than in their homes. This may reveal that 71% of professionals who reported never having identified a situation of VAOP may, in fact, have already been faced with a case of this type of violence, but were unable to identify it or did not investigate it. Corroborating these findings, another study reports that identifying cases is not easy. Therefore, it is pertinent that nurses have knowledge about the principles of forensic science, to provide adequate assistance when faced with a victim^(21)^.

Gaps in the management of these cases often result from a combination of factors, including lack of knowledge about protocols and lack of specialized training. The difficulties faced by professionals stand out, both in the early recognition of violence and in the articulation with support and protection networks, highlighting the need for standardization and implementation of clear protocols for dealing with VAOP^(22)^.

The care practice revealed in this study highlights a distance from the ideal advocated by public policies with regard to violence management. This highlights the urgent need to invest in training professionals and implementing flowcharts that guide care in different sectors. This initiative must be complemented by expanding spaces for intersectoral coordination and by creating an efficient institutional communication system between the various actors involved. This effort must overcome the discrepancy between the ideas conceived, the actions carried out, and the guidelines established^(23)^.

In addition to the above, it was found that most nurses in both groups refer or would refer cases of VAOPO to social services and other members of the multidisciplinary team. This approach is important so that each professional can act within their competence to resolve the situation. However, when it comes to referring the case to the police authorities and the Council for the Older People, the majority, in both groups, do not do so or would not do so, which goes against the ethical-legal recommendation determining that suspected or confirmed cases of VAOP must be mandatorily reported to these agencies^(24)^.

Regarding the performance of history taking to assess signs of violence, almost all nurses perform or would perform these actions, as well as provide qualified listening and support. These practices, if carried out with quality, allow the identification of violence and support the planning of care, since according to the literature, verbal and non-verbal responses are also evidence and therefore must be documented in nursing records^(25)^.

The VAOP record is present in most responses, but the majority do not or would not develop care plans for victims, nursing diagnoses, nor do they recognize potential victims. Furthermore, almost half of inexperienced nurses would not question the victim’s family, which reveals important gaps in the effective handling of cases. Healthcare professionals working in hospital units, especially in emergencies and ICUs, are more likely to encounter forensic situations, which requires them to be able to collect relevant data that guides care planning^(26)^, since it is part of the nurse’s practice.

Physical examination was performed in both groups. History taking, listening, embracement, and physical examination are essential in the profession, so that it is possible to carry out the nursing process, and should be part of the routine of every nurse, and, when it comes to suspected violence, it is recommended that the nurse conduct forensic nursing interviews and collect evidence through cephalopodal evaluation^(19)^. It is clear that the effectiveness of this assessment is important, because in addition to verbal and non-verbal aspects, the appearance and location of injuries can tell the story of physical trauma, including whether it is acute or chronic, in addition to other forms of mistreatment. Obtaining these details through physical examination, records, and appropriate imaging can provide evidence to corroborate or refute a suspicion of violence. Therefore, the quality of this assessment directly impacts the investigations^(27)^.

In addition, most participants revealed that they collaborate or would collaborate with the judiciary sector to resolve cases; however, when asked whether they preserve or would preserve traces, there was a low affirmative response, corroborating a study carried out in New Zealand, which revealed limitations in the knowledge of nurses to correctly identify forensic cases, especially with regard to the ethical-legal aspects inherent to clinical care, collection and preservation of traces^(19)^. Therefore, the need for nurses to preserve and collect evidence during the provision of care to allow legal cases to take place^(19)^ since, as revealed in another study, the chances of the victim reporting the violence increase 2.5 times when the injury is documented^(28)^.

Regarding interventions in cases of VAOP highlighted in this investigation, it was found that there are management gaps. Forensic interventions carried out by nurses, which are essential in the management of violence against older people, present weaknesses, especially in the collection of evidence and in the planning of care that guarantees safety and respect for the victim. Insufficient training and the absence of clear protocols compromise the proper management of cases^(5,19)^. Practices such as active listening, emotional support, and integration with support networks, although relevant, are still underused^(1,17)^.

In this study, it was found that the group with experience in VAOP carried out specific actions of Forensic Nursing. Among the interventions, the following stand out: collaboration with the judicial system, promotion of the protection of human rights and legal guarantees for victims, their families and the people who committed the violence, in addition to the preservation of traces in cases of mistreatment, sexual violence, and other forms of violence with the purpose of proving violence against older people. These practices reinforce the importance of the forensic nurse as an agent that integrates care, justice and science, promoting humanized assistance and contributing to the construction of a fairer and more effective health system^(21)^.

The limitations of this study include the difficulties presented by nurses in recognizing VAOP, since the professionals reported situations having characteristics of violence, without understanding that the verbalization of such attributes characterizes suspicion of the phenomenon. Furthermore, the study was carried out in a hospital context; therefore, it may not represent the reality of Primary Care. Based on this understanding, it is believed that the dissemination of the results of this research will enable scientific advances in health and nursing, thus contributing to guiding managers in the development of training aimed at expanding the skills of nurses who care for older people in situations of violence, and thus improving the care provided.

## CONCLUSION

The result of this study showed how hospital nurses, with and without experience in VAOP, intervene or will intervene, respectively, in these cases of violence. Comparing the two groups, significant similarity was found between both, with the majority of experienced nurses carrying out important interventions in the management of cases, despite carrying out essential actions with weaknesses, such as referring to the authorities, in addition to notifying support entities such as the Older People Council.

Thus, the need to use instruments and offer training to guide nursing care for older people is highlighted. Therefore, it is suggested that research and interventions be urgently carried out to improve nursing management regarding: referring the case to the police; developing a care plan and nursing diagnoses; questioning the older person’s family; recognizing situations of violence and preserving traces. These are essential actions to interrupt the cycle of violence, but which are managed with weaknesses. There is also the need to promote specialization in FN.
