# Supplementary material for: Forensic interventions carried out by nurses on older people in situations of violence: a comparative study
Source: Rev Esc Enferm USP. 2025 May 9;59:e20240282. doi: 10.1590/1980-220X-REEUSP-2024-0282en (PMC12068245; doi:10.1590/1980-220X-REEUSP-2024-0282en)
Supplement: Translation

# Translation: Intervenções forenses realizadas por enfermeiros à pessoa idosa em situação de violência: estudo comparativo*

**DOI:** 10.1590/1980-220X-REEUSP-2024-0282pt

**Authors:** Jiovana de Souza Santos, Francisca de Chagas Alves de Almeida, Jefferson da Silva Soares, Isabel Vitória do Nascimento Pereira Gomes, Raúl Fernando Guerrero Castañeda, Rafaella Queiroga Souto

**Affiliations:** 1 Universidade Federal do Pernambuco, Centro de Ciências da Saúde, Departamento de Enfermagem, Recife, PE, Brasil.; 2 Hospital Universitário Lauro Wanderley, João Pessoa, PB, Brasil.; 3 Universidade Federal da Paraíba, Centro de Ciências da Saúde, Curso de Enfermagem, João Pessoa, PB, Brasil.; 4 Universidade Federal da Paraíba, Centro de Ciências da Saúde, Curso de Graduação em Enfermagem, João Pessoa, PB, Brasil.; 5 Universidad de Guanajuato, Guanajuato, Gto, México.; 6 Universidade Federal da Paraíba, Centro de Ciências da Saúde, Departamento de Saúde Coletiva, João Pessoa, PB, Brasil.

**Keywords:** Atención de Enfermería, Enfermería Forense, Abuso de Ancianos, Violência, Nurses Improving Care for Health System Elders

## Abstract

**Objetivo:**

Investigar as intervenções forenses realizadas por enfermeiros com e sem experiência em atendimento à pessoa idosa em situação de violência.

**Método:**

Estudo transversal, quantitativo, com enfermeiros que atuam em cinco hospitais de João Pessoa, Paraíba, Brasil. A coleta de dados foi realizada por meio de um questionário estruturado, validado por dez especialistas e com boa fidedignidade, apresentando um coeficiente *Alfa de Cronbach* superior a 0,70. Os dados foram analisados por meio de frequência simples e relativa, teste Bayesiano para proporções, Teste *t-Student*, análise multivariada com modelos de análise de correspondência múltipla e de agrupamento com métrica Qui-Quadrado com forma de aglomeração hierárquica e método da ligação média.

**Resultados:**

Participaram 270 enfermeiros, dos quais 203 não tinham experiência com a pessoa idosa em situação de violência. Entre os grupos, verificou-se diferença significativa (P < 0,001), ou seja, as intervenções do grupo com a experiência são mais condizentes com os aspectos éticos-legais do que aquele sem experiência.

**Conclusão:**

Verificou-se semelhança significativa entre os dois grupos, em que enfermeiros com experiência realizaram intervenções adequadas e relevantes, embora a execução de ações para interromper o ciclo de violência apresentasse fragilidades.

## INTRODUÇÃO

A violência tem a hospitalização como significativa consequência e, nesse contexto, os profissionais de saúde possuem um papel relevante na identificação dos casos, uma vez que estão assistindo diretamente os usuários e têm a oportunidade, por vezes únicas, de identificar a situação. Porém, devido às fragilidades apresentadas por enfermeiros nas competências para atender pessoas idosas em situação de violência, respostas efetivas não têm sido alcançadas^(1)^. Nessa premissa, na última década, a enfermagem forense (EF) vem sendo expandida no Brasil, possibilitando a contribuição e o desempenho no processo de trabalho para que o enfermeiro possa desenvolver uma visão holística para identificar os casos de violência^(2)^, por meio da combinação da ciência de enfermagem, ciências forenses e os cuidados específicos de saúde.

A enfermagem forense é uma área especializada que combina a prática da enfermagem com conhecimentos clínicos e jurídicos, atendendo tanto vítimas, vivas ou falecidas, quanto infratores. Entre suas principais atribuições estão a detecção de sinais de violência por meio de anamnese e exame físico, a coleta, preservação e armazenamento de evidências, além da colaboração com o sistema judicial por meio de denúncias e depoimentos. Além disso, desempenha um papel fundamental ao oferecer assistência integral e humanizada às vítimas de violência^(3)^.

Nesse cenário, cabe ao enfermeiro identificar as lesões forenses, documentar, orientar, denunciar, coletar e preservar vestígios. Além disso, o princípio da confidencialidade deve ser respeitado e os dados da coleta de todas as informações sobre as evidências do crime preservados. Os pacientes forenses, além de cuidados físicos e psicológicos, ainda necessitam de proteção de seus direitos legais. Para assegurar esses direitos, é primordial o registro e coleta de evidências, além da preservação da cadeia de custódia^(4)^.

A literatura ressalta que qualquer evidência obtida aumenta as taxas de acusação e condenação por crimes, o que faz com que os perpetradores recebam sentenças mais longas e, principalmente, que isso resulte na quebra do ciclo da violência^(5)^. Nessa perspectiva, o Conselho Federal de Enfermagem (COFEN) destaca que o enfermeiro possui uma compreensão do sistema sanitário, social e jurídico, enriquecido pelo conhecimento das ciências forenses e da saúde pública, podendo atuar junto ao sistema criminal, agentes do governo e interpretações sobre lesões forenses^(6)^.

O conceito de lesão forense pode ser definida como dano causado à integridade física de uma pessoa, sendo constituída por qualquer alteração na estrutura ou na função do corpo humano resultante de fatores externos, cuja análise é realizada para fins de investigação judicial, identificando a causa, a extensão do dano e o instrumento utilizado^(7)^.

Nesse contexto, a intervenção forense é um processo que envolve a aplicação de conhecimentos técnicos e científicos em investigações que exigem esclarecimentos de natureza jurídica, com o objetivo de subsidiar a Justiça. Assim, busca fornecer elementos técnicos para a tomada de decisão por autoridades judiciais^(7)^. Essa intervenção ocorre de forma interdisciplinar e, no tocante à enfermagem, podem-se citar como exemplos a recepção acolhedora, a escuta ativa, o registro de lesões físicas, a coleta de vestígios biológicos, como sêmen, e a preservação da integridade das provas^(8)^.

A atuação do enfermeiro frente à violência perpassa por todos os gêneros e faixas etárias; no entanto, este estudo dá ênfase à violência contra a pessoa idosa (VCPI), a qual é considerada um problema de saúde pública que exige intervenções efetivas. No Brasil, um estudo multicêntrico realizado em dois hospitais, com 323 idosos, apontou que mais da metade dos participantes apresentaram risco de violência^(9)^.

Assim, revelar as potencialidades e as fragilidades da atuação de enfermeiro acerca das nuances que envolvem o manejo da VCPI é necessário, uma vez que isto mostra a realidade e permite intervenções. Desse modo, preparar esse profissional para conduzir os casos de violência é considerar que seu trabalho envolve uma observação cuidadosa da vida diária da pessoa idosa, dando-lhes a oportunidade de prevenir, detectar e interromper a ação^(10)^.

Diante do exposto surgem as seguintes questões: como o enfermeiro intervém ou intervirá em casos de VCPI no contexto hospitalar? Há diferenças nas intervenções entre enfermeiros que atenderam e não atenderam os casos de VCPI? Para responder aos questionamentos, objetivou-se investigar as intervenções forenses realizadas por enfermeiros com e sem experiência em atendimento à pessoa idosa em situação de violência. Assim, a realização desse estudo justifica-se ao considerar que pesquisas sobre intervenções de enfermeiros na VCPI em hospitais ainda é escassa e evidenciar as fragilidades e potencialidades dessa atuação são imprescindíveis para a tomada de decisão.

## MÉTODO

### Tipo do Estudo

Trata-se de um estudo descritivo transversal, abordagem quantitativa, conduzido com base nas recomendações da ferramenta *STrengthening the Reporting of OBservational studies in Epidemiology* (STROBE).

### Local do Estudo

O estudo foi realizado em cinco hospitais públicos de João Pessoa, PB, Brasil, selecionados por atenderem uma quantidade significativa de pessoas idosas, nos setores: urgência, clínica médica, clínica cirúrgica e Unidade de Terapia Intensiva (UTI).

### População e Critérios de Seleção

A população foi composta por 660 enfermeiros, dos quais 285 estavam distribuídos nos quatro setores selecionados. Os critérios de elegibilidade foram: ser enfermeiros assistenciais, com no mínimo quatro semanas de atuação no setor e 20 horas semanais de trabalho. Considerou-se esse período mínimo por ser o necessário para que o profissional seja exposto a situações práticas inerentes à sua profissão, o que permite responder com eficácia à pesquisa^(11)^. Foram excluídos os enfermeiros que desempenham atividades exclusivamente gerenciais e aqueles que estivessem afastados do trabalho, por qualquer motivo.

### Definição da Amostra

Considerando a população acessível como sendo todos os enfermeiros dos quatro setores, tem-se uma população finita de tamanho N = 285. Assim, uma amostra aleatória simples com 98% de confiança e margem de erro de 2% foi considerada. Obteve-se o tamanho da amostra com a aplicação da fórmula abaixo, onde z = 1,96; P = 0,50; Q = 1−P; N = 285 e d = 0,03 e com correção de população finita com a expressão n_f_ ≈ n / [1+ n /N]^(12)^. Assim, foram necessárias 253 para amostragem mínima, porém 270 foram coletadas.

$$n=\frac{\frac{z^{2}PQ}{d^{2}}}{1+\frac{1}{N}\left(\frac{z^{2}PQ}{d^{2}}-1\right)}$$

### Coleta de Dados

A coleta ocorreu de junho a dezembro de 2022, por meio de um questionário estruturado desenvolvido pela pesquisadora e validado por 10 expertises. Para aplicação do instrumento junto aos participantes, uma abordagem por conveniência foi realizada, indo-se ao encontro aos profissionais em horários disponíveis. O pesquisador realizou visitas semanais aos setores das instituições, convidando os enfermeiros em atividade a participarem. O questionário foi aplicado no local indicado pelos próprios participantes, seguindo rigorosamente o protocolo de uso de máscara cirúrgica, em conformidade com as medidas de prevenção da Covid-19.

Para a elaboração do questionário, fez-se uma revisão de escopo “Cuidado de enfermagem forense ao idoso em situações de violência: revisão de escopo” com objetivo de identificar como esse cuidado ocorre. Os itens do instrumento apresentaram boa fidedignidade com *Alpha de Cronbach* superior a 0,70, considerado uma boa medida^(13)^, mostrando que o instrumento permite identificar as intervenções dos enfermeiros frente à VCPI. Além disso, o coeficiente de correlação do ponto bisserial entre cada item apresenta valores abaixo de 0,50, mostrando que todos os itens do instrumento são importantes. A probabilidade de o participante ter “chutado” uma resposta, acerto ao acaso, foi menor que 0,04, sendo aceitável até 0,05^(14)^.

O questionário foi composto por dados sociodemográficos e aspectos gerais da VCPI, contemplando 11 itens, identificados pela letra “A” seguida de um número arábico do item; e por ações realizadas pelos enfermeiros nos casos de VCPI, com 16 itens, identificados pela letra “B” e sequência de número arábico com respostas “sim”, “não” e “talvez”. Para aplicar o instrumento, foi realizada abordagem por conveniência, indo-se ao encontro dos profissionais, levando em consideração sua disponibilidade.

### Análise e Tratamento dos Dados

Os dados foram organizados em uma planilha do *Excel* (Microsoft 365, 2019) e a análise realizada com o *software* livre Jamovi Versão 0.9, 2020 e SPSS (*Statistical Package for the Social Sciences*) versão 20.0. Para caracterização dos dados sociodemográficos e da VCPI, utilizou-se a frequência simples e relativa. Em relação à distinção dos grupos com e sem experiência, aplicou-se o teste da proporção. No caso de impossibilidade da aplicação desse teste (proporções próximas de 0 ou 1), utilizou-se a comparação por meio do teste Bayesiano para proporções. Para associação entre variáveis quantitativas, foi realizado o Teste *t-Student* e para diferenciação dos itens de maior e menor porcentagens de respostas utilizou-se a análise estatística multivariada aplicando-se a Análise de Correspondência Múltipla (ACM) com apresentação dos resultados em mapa perceptual e análise de agrupamento com a métrica Qui-Quadrado, com forma de aglomeração hierárquica e método da ligação média em dendograma^(14,15)^. A consistência interna do instrumento foi analisada com o *Alfa de Cronbach*, com intervalo de 95% de confiança.

### Aspectos Éticos

Essa pesquisa faz parte do projeto Instrumentalização da enfermagem forense diante do cuidado ao idoso hospitalizado, aprovada pelo Comitê de Ética em Pesquisa sob parecer nº 3.709.600 e CAAE nº10179719.9.0000.5183, seguindo a Resolução 466/2012 do Conselho Nacional de Saúde.

## RESULTADOS

Participaram da pesquisa 270 enfermeiros. Desses, 203 (75%) não tinham experiência com VCPI e 67 (25%) tinham. Observa-se, nos dois grupos – com e sem experiência em VCPI, que a maioria dos participantes são mulheres (236 – 87,4%), na faixa etária de 31 a 40 anos (172 – 63,8%), especialistas (244 – 90,4%), atuantes no setor de urgência (103 – 38,1%) e clínica médica (69 – 25,6%) com pelo menos dois empregos (204 – 75,5%). Quanto ao tempo de serviço, os participantes sem experiência em VCPI tinham mais de dez anos de atuação no setor (60 – 29,5%) enquanto 30 (44,7%) entre os participantes com experiência em VCPI tinham de três a seis anos de atuação no setor, conforme Tabela 1.

**Tabela 1:** Variáveis sociodemográficas dos enfermeiros – João Pessoa, PB, Brasil, 2022.

Variável	Categoria	Sem		Com		Total	
		N	%	N	%	N	%
**Sexo**	Masculino	24	8,8	10	3,7	34	12,6
	Feminino	179	66,2	57	21,1	236	87,4
**Idade**	18 a 30	4	1,5	14	5,1	18	6,7
	31 a 40	131	48,5	41	15,1	172	63,8
	41 a 50	67	24,8	8	2,9	75	27,8
	60 a 70	1	0,4	4	1,5	5	1,9
**Escolaridade**	Doutorado	2	0,7	1	0,3	3	1,1
	Mestrado	5	1,8	4	1,5	9	3,3
	Especialista	188	69,6	56	20,7	244	90,4
	Graduado	8	2,9	6	2,2	14	5,2
**Setor**	Clínica Cirúrgica	38	14,0	10	3,7	48	17,8
	Clínica Médica	40	14,8	29	10,7	69	25,6
	Urgência	89	32,9	14	5,1	103	38,1
	UTI	36	13,3	14	5,1	50	18,5
**Quantidade de empregos**	Um	7	2,6	21	7,8	28	10,4
	Dois	165	61,1	39	14,4	204	75,5
	Três	30	11,1	7	2,59	37	13,7
	Quatro	1	0,3	0	0,0	1	0,3
**Tempo de atuação no setor (em anos)**	≤1	10	4,9	5	7,4	15	5,6
	1 a 3	55	27,0	15	22,3	70	25,9
	3 a 6	38	18,7	30	44,7	68	25,2
	6 a 9	40	19,7	7	10,4	47	17,4
	≤10	60	29,5	10	14,9	70	25,9

Em relação aos aspectos gerais acerca da VCPI, apenas no item A5 observou-se semelhança nas respostas entre os dois grupos, e diferença significativa p < 0,005 nos demais itens como mostra a Tabela 2.

**Tabela 2:** Aspectos gerais acerca da VCPI considerando as respostas dos dois grupos – João Pessoa, PB, Brasil, 2022.

Item	Categoria	Sem		Com		Total		Valor-P teste de Fisher
		N	%	N	%	N	%	
A1 – Já identificou algum caso ou suspeita de Violência contra a pessoa idosa?	**Sim**	0	0,0	67	100	67	24,8	
	**Não**	198	97,5	0	0,0	198	73,3	< 0,001*
	**Talvez**	5	2,5	0	0,0	5	1,9	
A2 – Já percebeu algum desconforto do idoso na presença dos seus familiares?	**Sim**	74	36,5	62	92,5	136	50,4	
	**Não**	128	63,1	4	6,0	132	48,9	< 0,001*
	**Talvez**	1	0,5	1	1,5	2	0,7	
A3 – Acha fácil detectar que o idoso está em situação de violência?	**Sim**	35	17,2	19	28,4	54	20,0	
	**Não**	165	81,3	42	62,7	207	76,7	0,0014*
	**Talvez**	3	1,5	6	9,0	9	3,3	
A4 – Conhece algum instrumento ou escala que ajude na assistência prestada ao idoso com suspeita ou confirmação de violência? (mesmo que nunca tenha utilizado)	**Sim**	2	1,0	9	13,4	11	4,1	
	**Não**	201	99,0	56	83,6	257	95,2	
	**Talvez**	0	0,0	2	3,0	2	0,7	< 0,001*
A5 – Considera importante ter algum material (protocolo, escala, instrumento) para auxiliar no cuidado ao idoso vítima de violência?	**Sim**	201	99,0	66	68,5	267	98,9	
	**Não**	2	1,0	1	1,5	3	1,1	0,9999*
	**Talvez**	0	0,0	0	0,0	0	0,0	
A6 – Utiliza algum instrumento ou escala que ajude na assistência prestada ao idoso com suspeita ou confirmação de violência?	**Sim**	1	0,8	5	7,5	6	2,2	
	**Não**	201	99,5	60	89,5	262	97,0	
	**Talvez**	0	0,0	2	3,0	2	0,7	0,0004*
A7 – Sente-se preparada (o) para identificar situações de violência contra a pessoa idosa?	**Sim**	63	31	11	16,4	74	27,4	
	**Não**	133	65,5	47	70,1	180	66,7	0,0026*
	**Talvez**	7	3,5	9	13,4	16	5,9	
A8 – Acha que há oferta de capacitação suficiente para os enfermeiros identificarem situações de violência contra o idoso?	**Sim**	0	0,0	7	10,4	7	2,6	
	**Não**	203	100	59	88,1	262	97,0	
	**Talvez**	0	0,0	1	1,5	1	0,4	< 0,001*
A9 – Acha que os idosos escondem que sofreram violência?	**Sim**	146	71,9	62	92,5	234	86,7	
	**Não**	57	77,3	3	4,5	33	12,3	< 0,001*
	**Talvez**	0	0,0	2	3,0	3	1,1	
A10 – Já presenciou situações em que o idoso relata ser mais bem cuidado no hospital do que em seu ambiente familiar?	**Sim**	146	71,9	62	92,5	108	40	
	**Não**	57	28,0	3	4,5	160	59,3	
	**Talvez**	0	0,0	2	3,0	2	0,7	< 0,001*
A11 – Considera que a violência prejudica a melhora do idoso quando ele se encontra internado no hospital?	**Sim**	172	84,7	62	92,5	234	86,7	
	**Não**	31	15,3	3	4,5	3	1,1	
	**Talvez**	0	0,0	2	3,0	33	12,2	0,0033*

*Teste de Fisher. A = categorização do item.

Na tabela 3, observa-se que há diferença significativa na maioria dos itens em ambos os grupos, exceto nos itens B1 e B5. Ao final da tabela 3, o teste *t-Student,* adequado para grandes amostras na comparação de médias, confirma que há diferença significativa entre os escores obtidos pelos dois grupos.

**Tabela 3:** Comparação das intervenções dos enfermeiros com e sem a experiência em VCPI – João Pessoa, PB, Brasil, 2022.

Item	Categoria	Sem			Com		Total		Valor-P Qui-quadrado
		N	%		N	%	N	%	
B1 – Encaminhou ou encaminharia para Assistente Social os casos suspeitos ou confirmados?	**Sim** **Não** **Talvez**	203 0 0	100 0 0		65 2 0	97,0 2,9 0	268 2 0	98,9 0,7 0,0	0,3075
B2 – Encaminhou ou encaminharia para autoridade policial os casos suspeitos ou confirmados?	**Sim** **Não** **Talvez**	11 189 3	5,4 93,1 1,5		26 38 3	38,8 56,7 4,4	37 225 8	13,7 83,6 2,9	< 0,001*
B3 – Encaminhou ou encaminharia para o Conselho do idoso os casos suspeitos ou confirmados?	**Sim** **Não** **Talvez**	13 189 1	6,4 93,1 0,5		26 40 4	38,8 59,7 6	39 224 7	14,4 83,3 2,6	< 0,001*
B4 – Realiza anamnese/entrevista no idoso para avaliar sinais e sintomas de violência?	**Sim** **Não** **Talvez**	201 1 0	99,0 0,5 0,0		59 7 1	88,1 10,4 1,5	260 7 3	95,9 2,6 2,1	< 0,001*
B5 – Realiza uma escuta qualificada para tentar identificar os casos suspeitos de violência contra o idoso?	**Sim** **Não** **Talvez**	171 30 1	83,3 14,7 0,5		60 4 3	89,6 5,9 4,4	229 35 6	84,8 12,9 2,2	0,3429
B6 – Realiza exame físico no idoso para avaliar sinais e sintomas de violência?	**Sim** **Não** **Talvez**	112 46 30	54,9 22,6 14,7		61 4 2	91,0 5,9 3	173 50 32	64,0 18,5 11,8	< 0,001*
B7 – Registra ou registraria em prontuário os casos suspeitos ou confirmados?	**Sim** **Não** **Talvez**	201 2 0	98,5 0,9 0,0		58 4 5	86,6 5,9 7,4	259 5 6	95,9 1,8 2,2	< 0,001*
B8 – Elabora ou elaboraria planos de cuidados para o idoso e famílias envolvidas em situações de maus tratos, abuso sexual, traumas e outras formas de violência?	**Sim** **Não** **Talvez**	42 160 1	20,6 78,8 0,5		27 39 1	40,3 58,2 1,5	69 199 2	25,5 73,7 0,7	0,0023*
B9 – Acolhe os idosos vítimas de violência sexual, traumas e outras formas de violência?	**Sim** **Não** **Talvez**	203 0 0	100 0,0 0,0		63 3 1	94,0 4,5 1,5	266 4 0	98,5 1,5 0,0	0,0178*
B10 – Questiona a família do idoso no caso de suspeita ou confirmação?	**Sim** **Não** **Talvez**	103 52 40	50,7 25,6 19,7		57 5 5	85,1 7,4 7,4	160 57 45	59,2 21,1 16,6	< 0,001*
B11 – Comunica a equipe multiprofissional os casos de suspeita ou confirmação de violência contra o idoso para tentar solucionar o problema?	**Sim** **Não** **Talvez**	203 0 0	100 0,0 0,0		61 5 1	91,0 7,4 1,5	265 4 1	98,1 1,4 0,3	0,667B**
B12 – Em caso suspeito ou confirmado de violência contra o idosos, comunica ao médico a situação para ajudá-la na condução.	**Sim** **Não** **Talvez**	203 0 0	100 0,0 0,0		62 4 1	92,5 1,4 1,5	265 4 1	98,1 1,4 0,3	0,130B**
B13 – Colabora com o sistema judiciário nos casos necessários.	**Sim** **Não** **Talvez**	171 30 2	83,8 41,2 0,9		59 5 3	88,1 7,4 4,4	230 34 6	85,1 12,6 2,2	0,0039*
B14 – Reconhece possíveis situações de violência contra o idoso, identifica potenciais vítimas e elabora diagnósticos de enfermagem no contexto de maus tratos, traumas, violência sexual e outras formas de violência.	**Sim** **Não** **Talvez**	10 192 1	4,9 94,5 0,5		36 27 4	53,7 40 6	46 216 8	17,0 80 2,9	< 0,001*
B15 – Promove ou promoveria a proteção dos direitos humanos e das garantias legais do idoso, das suas famílias e das pessoas que cometeu a violência.	**Sim** **Não** **Talvez**	196 2 5	96,1 0,9 2,4		59 3 5	88,1 4,5 7,4	255 6 9	94,4 2,2 3,3	0,0340*
B16 – Preserva ou preservaria vestígios em casos de maus-tratos, violência sexual e outras formas de violência para fins de comprovação da violência exercida contra o idoso.	**Sim** **Não** **Talvez**	13 187 3	6,4 92,1 1,4		27 38 2	40,2 56,7 2,9	54 208 8	20 77,0 2,9	< 0,001*
**Média**		10,0			12,2		10,6		**Valor-P**
**Mediana**		11,0			12,0		11,0		0,0020*t
**Desvio Padrão**		1,7			3,0		2,3		

*Diferença significativa em nível de 5% – Teste Qui-quadrado.

**B = Utilizou teste Bayesiano de duas proporções.

*t = Teste t-Student. B = categorização do item.

Verifica-se que há itens com valores de percentuais de resposta elevados e outros muito baixos na tabela 3, e para decidir quais são os itens diferenciados com alguma destas caracterizações utilizou-se a análise estatística multivariada, aplicando-se os modelos de ACM^(15)^ e análise de agrupamento^(16)^.

Os resultados da ACM apresentados na Tabela 4 permitem concluir que uma única dimensão de itens da tabela 3 responde por 98,4% da variabilidade total contida nas informações da Tabela 3. Portanto, a dimensão 2 (itens: B1, B4, B5, B7, B9, B11, B12, B15) apresenta itens mais associados a respostas afirmativas.

**Tabela 4:** Análise de Correspondência Múltipla das respostas da tabela 3 – João Pessoa, PB, Brasil, 2022.

Dimensão	Valor singular	Inércia	Qui-quadrado*	Valor-P	% Inércia
1	0,096	0,009	2379,93	< 0,001	1,6
2	0,740	0,548			98,4
Total		0,557			100,0

Na Figura 1, tem-se o mapa perceptual em duas dimensões, onde se percebe que há três aglomerados cujo significado, na ACM, é que quanto maior a proximidade entre os níveis das categorias da Tabela 3 maior é a associação entre estes elementos formadores do aglomerado^(16)^. O maior aglomerado é formado pela associação entre os itens B1, B4, B5, B7, B9, B11, B12, B13 e B15 e a resposta “sim”.

**Figura 1:** Mapa perceptual da ACM e Dendograma da Análise de Agrupamento da Tabela 3 – João Pessoa, PB, Brasil, 2022.

Percebe-se que a característica comum a todos esses itens é seu elevado percentual de ocorrência. Os itens B6 e B10 estão mais associados às respostas “talvez” para o grupo sem experiência na VCPI e, finalmente, o aglomerado da direita formado pelos itens B2, B3, B8, B14 e B16, é o que apresenta itens com os menores percentuais de respostas “sim”, estando mais associados às respostas “não” para ambos os grupos.

A análise de Agrupamento com a métrica Qui-Quadrado com forma de aglomeração hierárquica e método da ligação média^(15)^ tem seu dendrograma apresentado ao lado do mapa perceptual, e observa-se um corte vertical na distância igual a 10, onde se observam os mesmos grupos de itens apresentados na ACM, confirmando a mesma interpretação dada aos resultados apresentados no mapa perceptual.

## DISCUSSÃO

Revelou-se que, em relação aos aspectos gerais da VCPI, a maioria dos enfermeiros, em ambos os grupos, já percebeu desconforto da pessoa idosa na presença de familiar, achou difícil detectar a VCPI, refere não conhecer e não utilizar instrumentos e/ou protocolos que auxiliem na identificação e intervenção dos casos e acha importante tê-los. Além disso, os enfermeiros não se sentem preparados para identificarem a VCPI, relatam que não há oferta de capacitações suficientes para prepará-los, o que dificulta a condução dos casos, uma vez que a pessoa idosa esconde a violência.

Em consonância com os achados, estudos sobre as percepções dos profissionais de enfermagem em relação à detecção e prevenção da VCPI revelam que, embora muitos considerem ou suspeitem da existência de casos, enfrentam dificuldades para agir, especialmente devido à falta de treinamento específico e à ausência de integração entre os serviços públicos direcionados a esse tipo de cuidado. Além disso, demonstram preocupação quanto à ausência de preparo^(17,18)^.

Diante da ausência de preparo revelado no presente estudo e em outras pesquisas, é importante ressaltar que os enfermeiros exercem papel significativo nas implicações legais dos casos forenses e, para isso, necessitam adquirir conhecimentos da enfermagem em ciências forenses^(19)^. Além disso, o uso de protocolos e instrumentos que norteiam a condução da VCPI são fundamentais para uma assistência adequada^(20)^.

Nessa pesquisa, percebeu-se que a maioria dos enfermeiros revelou que já presenciou a pessoa idosa relatar que é mais bem cuidada no ambiente hospitalar do que na sua residência. Isso pode revelar que 71% dos profissionais que referiram nunca ter identificado uma situação de VCPI podem, na verdade, já ter estado diante de um caso desse tipo de violência, mas não souberam identificar ou não investigaram. Corroborando esses achados, outra pesquisa relata que a identificação dos casos não é fácil. Por isso, é pertinente que os enfermeiros tenham conhecimento acerca dos princípios das ciências forenses, para prestarem assistência adequada ao deparar-se com uma vítima^(21)^.

As lacunas no manejo desses casos muitas vezes resultam de uma combinação de fatores, incluindo desconhecimento sobre protocolos e ausência de formação especializada. Destacam-se as dificuldades enfrentadas pelos profissionais, tanto no reconhecimento precoce da violência, quanto na articulação com redes de apoio e proteção, evidenciando a necessidade de padronização e implementação de protocolos claros para lidar com a VCPI^(22)^.

A prática assistencial revelada no presente estudo evidencia um distanciamento em relação ao ideal preconizado pelas políticas públicas no que diz respeito à gestão da violência. Isso destaca a necessidade urgente de investir na capacitação dos profissionais e na implementação de fluxogramas que orientam o cuidado em diferentes setores. Essa iniciativa deve ser complementada pela ampliação de espaços para articulação intersetorial e pela criação de um sistema institucional de comunicação eficiente entre os diversos atores envolvidos. Esse esforço deve superar o descompasso entre as ideias concebidas, as ações realizadas e as diretrizes estabelecidas^(23)^.

Somado ao exposto, constatou-se que a maior parte dos enfermeiros de ambos os grupos encaminham ou encaminhariam os casos de VCPI ao serviço social e demais membros da equipe multiprofissional. Essa condução faz-se importante para que cada profissional atue dentro da sua competência para a resolutividade da situação. Porém, quando se trata de encaminhamento do caso à autoridade policial e Conselho da Pessoa Idosa, a maioria, nos dois grupos, não o faz ou não fariam, o que vai contra a recomendação ético-legal, a qual determina que os casos suspeitos ou confirmados de VCPI devem ser obrigatoriamente comunicados a esses órgãos^(24)^.

No tocante à realização de anamnese para avaliar sinais de violência, quase a totalidade dos enfermeiros executa ou executaria essas ações, bem como também realiza escuta qualificada e acolhimento. Essas práticas, se realizadas com qualidade, permitem identificar a violência e subsidiar o planejamento da assistência, pois conforme a literatura, as respostas verbais e não verbais também são evidências e por isso devem ser documentadas nos registros de enfermagem^(25)^.

O registro de VCPI está presente na maior parte das respostas, mas essa maioria não elabora ou elaboraria planos de cuidados às vítimas, diagnósticos de enfermagem e tampouco reconhece potenciais vítimas. Além disso, quase metade dos enfermeiros sem experiência não questionaria a família da vítima, o que revela lacunas importantes para a condução efetiva dos casos. Profissionais de saúde que atuam em unidades hospitalares, principalmente em emergências e UTI, têm maior probabilidade de deparar-se com situações forenses, o que exige deles capacidade para coletar dados pertinentes que conduzam o planejamento do cuidado^(26)^, uma vez que faz parte do exercício do enfermeiro.

A realização de exame físico foi constatado nos dois grupos. A anamnese, escuta, acolhimento e exame físico são essenciais na atuação da profissão, para que assim, seja possível executar o processo de enfermagem, devendo fazer parte da rotina de todo enfermeiro, e, quando se tratar de suspeita de violência, recomenda-se que o enfermeiro conduza entrevistas de enfermagem forense e colete evidências por meio de avaliação céfalo-podal^(19)^. Constata-se que a efetividade dessa avaliação é importante, pois além dos aspectos verbais e não verbais, a aparência e a localização de lesões podem contar a história de trauma físico, inclusive se é agudo ou crônico, além de outros maus-tratos. A obtenção desses detalhes por meio de exame físico, registros e imagens adequadas podem fornecer evidências para corroborar ou refutar uma suspeita de violência. Portanto, a qualidade dessa avaliação impacta diretamente nas investigações^(27)^.

Ademais, a maioria dos participantes revelou que colabora ou colaboraria com o poder judiciário para resolução dos casos; no entanto, quando questionados se preservam ou preservariam vestígios, houve baixa resposta afirmativa, corroborando estudo realizado na Nova Zelândia, que revelou limitação no conhecimento de enfermeiros para identificar corretamente os casos forenses, principalmente no que se refere aos aspectos éticos-legais inerentes ao atendimento clínico, coleta e preservação de vestígios^(19)^. Diante disso, enfatiza-se a necessidade de os enfermeiros preservarem e coletar evidências durante a prestação de cuidados para permitir que os processos legais ocorram^(19)^ uma vez que, como revelado em outro estudo, as chances de a vítima relatar a violência aumentam 2,5 vezes quando a lesão é documentada^(28)^.

No que tange às intervenções dos casos de VCPI evidenciados nesta investigação, verificou-se que há lacunas no manejo. As intervenções forenses realizadas por enfermeiros, essenciais no manejo da violência contra a pessoa idosa, apresentam fragilidades, sobretudo na coleta de evidências e no planejamento de cuidados que garantam segurança e respeito à vítima. A insuficiência de capacitação e a ausência de protocolos claros comprometem a condução adequada dos casos^(5,19)^. Práticas como escuta ativa, suporte emocional e integração com redes de apoio, embora relevantes, ainda são subutilizadas^(1,17)^.

Neste estudo foi constatada a realização, pelo grupo com experiência em VCPI, de ações específicas da Enfermagem Forense. Dentre as intervenções, destacam-se a colaboração com sistema judiciário, promoção da proteção dos direitos humanos e das garantias legais das vítimas, das suas famílias e das pessoas que cometeram a violência, além da preservação de vestígios em casos de maus-tratos, violência sexual e outras formas de violência para fins de comprovação da violência exercida contra o idoso. Essas práticas reforçam a importância do enfermeiro forense como agente que integra cuidado, justiça e ciência, promovendo assistência humanizada e contribuindo para a construção de um sistema de saúde mais justo e efetivo^(21)^.

Ressaltam-se como limitações desse estudo as dificuldades apresentadas por enfermeiros para reconhecerem a VCPI, visto que os profissionais referiam situações características de violência, sem compreender que a verbalização de tais atributos caracteriza suspeita do fenômeno. Ademais, o estudo foi realizado no contexto hospitalar; logo, pode não representar a realidade da Atenção Básica A partir desse entendimento, acredita-se que a divulgação dos resultados dessa pesquisa possibilitará avanços científicos à saúde e à enfermagem, desse modo contribuindo para nortear gestores na elaboração de capacitações voltadas à ampliação das competências de enfermeiros que atendem pessoas idosas em situação de violência, e assim melhorar a assistência prestada.

## CONCLUSÃO

O resultado deste estudo mostrou como os enfermeiros de hospitais, com e sem a experiência em VCPI, intervêm ou intervirão, respectivamente, nesses casos de violência. Comparando os dois grupos, verificou-se semelhança significativa entre ambos, sendo que a maioria dos enfermeiros com experiência realizou intervenções importantes na condução dos casos, apesar de executar ações essenciais com fragilidades, tais como encaminhar às autoridades, além de notificar entidades de apoio como o Conselho de idosos.

Desse modo, destaca-se a necessidade do uso de instrumentos e oferta de capacitações para nortear a assistência de enfermeiros à pessoa idosa. Assim, sugere-se que seja pressurosa a realização de pesquisas e intervenções para melhorar o manejo de enfermeiros acerca de: encaminhamento do caso à polícia; elaboração de plano de cuidado e diagnósticos de enfermagem; questionamento à família da pessoa idosa; reconhecimento das situações de violência e preservação de vestígios, ações primordiais para interromper o ciclo de violência, mas que são manejadas com fragilidades. Acrescenta-se ainda a necessidade da divulgação da especialização em EF.

# Translation: Intervenciones forenses realizadas por enfermeros a personas mayores en situación de violencia: estudio comparativo*

**DOI:** 10.1590/1980-220X-REEUSP-2024-0282es

**Authors:** Jiovana de Souza Santos, Francisca de Chagas Alves de Almeida, Jefferson da Silva Soares, Isabel Vitória do Nascimento Pereira Gomes, Raúl Fernando Guerrero Castañeda, Rafaella Queiroga Souto

**Affiliations:** 1 Universidade Federal do Pernambuco, Centro de Ciências da Saúde, Departamento de Enfermagem, Recife, PE, Brasil.; 2 Hospital Universitário Lauro Wanderley, João Pessoa, PB, Brasil.; 3 Universidade Federal da Paraíba, Centro de Ciências da Saúde, Curso de Enfermagem, João Pessoa, PB, Brasil.; 4 Universidade Federal da Paraíba, Centro de Ciências da Saúde, Curso de Graduação em Enfermagem, João Pessoa, PB, Brasil.; 5 Universidad de Guanajuato, Guanajuato, Gto, México.; 6 Universidade Federal da Paraíba, Centro de Ciências da Saúde, Departamento de Saúde Coletiva, João Pessoa, PB, Brasil.

**Keywords:** Cuidados de enfermería, Enfermería forense, Maltrato a ancianos, Violencia, Cuidados de enfermería a ancianos hospitalizados

## Abstract

**Objetivo:**

Investigar las intervenciones forenses realizadas por enfermeros con y sin experiencia en el cuidado de ancianos en situaciones de violencia.

**Método:**

Se trató de un estudio cuantitativo transversal de enfermeros que trabajan en cinco hospitales de João Pessoa, Paraíba, Brasil. Los datos se recogieron mediante un cuestionario estructurado, validado por diez especialistas y con buena fiabilidad, mostrando un coeficiente alfa de Cronbach superior a 0,70. Los datos se analizaron mediante frecuencias simples y relativas, prueba bayesiana de proporciones, prueba t de Student, análisis multivariante con análisis de correspondencias múltiples y modelos de agrupación mediante la métrica chi-cuadrado con agrupación jerárquica y el método average linkage.

**Resultados:**

Participaron 270 enfermeras, 203 de las cuales no tenían experiencia con personas mayores en situaciones de violencia. Hubo una diferencia significativa entre los grupos (P < 0,001), es decir, las intervenciones del grupo con experiencia se ajustaron más a los aspectos ético-legales que las del grupo sin experiencia.

**Conclusión:**

Hubo similitud significativa entre los dos grupos, en el sentido de que las enfermeras con experiencia realizaron intervenciones adecuadas y pertinentes, aunque la implementación de acciones para interrumpir el ciclo de violencia mostró debilidades.

## INTRODUCCIÓN

La violencia tiene como consecuencia significativa la hospitalización y, en este contexto, los profesionales de la salud desempeñan un papel importante en la identificación de los casos, ya que atienden directamente a los usuarios y tienen la oportunidad, a veces única, de identificar la situación. Sin embargo, debido a la debilidad de las enfermeras en sus competencias para atender a personas mayores en situaciones de violencia, no se han conseguido respuestas eficaces^(1)^. Partiendo de esta premisa, la enfermería forense (EF) se ha expandido en Brasil en la última década, capacitando a las enfermeras para contribuir y actuar en el proceso de trabajo, de modo que puedan desarrollar una visión holística para identificar los casos de violencia^(2)^, combinando la ciencia de la enfermería, la ciencia forense y los cuidados específicos de salud.

La enfermería forense es un área especializada que combina la práctica de la enfermería con los conocimientos clínicos y jurídicos, atendiendo tanto a víctimas vivas como fallecidas y a agresores. Sus principales tareas incluyen la detección de signos de violencia a través de la anamnesis y la exploración física, la recogida, conservación y almacenamiento de pruebas, así como la colaboración con la justicia a través de denuncias y testimonios. También desempeña un papel fundamental al ofrecer una atención integral y humanizada a las víctimas de la violencia^(3)^.

En este escenario, las enfermeras son responsables de identificar las lesiones forenses, documentar, asesorar, informar, recoger y preservar los rastros. Además, se debe respetar el principio de confidencialidad y preservar los datos procedentes de la recogida de toda la información sobre las pruebas del delito. Además de la atención física y psicológica, los pacientes forenses también necesitan que se protejan sus derechos legales. Para garantizar estos derechos, es esencial registrar y recoger las pruebas, así como preservar la cadena de custodia^(4)^.

La literatura destaca que cualquier prueba obtenida aumenta las tasas de procesamiento y condena de los delitos, lo que significa que los autores reciben penas más largas y, sobre todo, que esto se traduce en la ruptura del ciclo de la violencia^(5)^. Desde esta perspectiva, el Consejo Federal de Enfermería (COFEN) destaca que las enfermeras tienen una comprensión del sistema sanitario, social y jurídico, enriquecida por el conocimiento de las ciencias forenses y de la salud pública, y pueden trabajar con el sistema de justicia penal, los agentes gubernamentales y las interpretaciones de las lesiones forenses^(6)^.

El concepto de lesión forense puede definirse como el daño causado a la integridad física de una persona, consistente en cualquier alteración en la estructura o función del cuerpo humano resultante de factores externos, cuyo análisis se realiza con fines de investigación judicial, identificando la causa, la extensión del daño y el instrumento utilizado^(7)^.

En este contexto, la intervención forense es un proceso que involucra la aplicación de conocimientos técnicos y científicos en investigaciones que requieren esclarecimiento de naturaleza jurídica, con el objetivo de subsidiar la justicia. Así, busca proporcionar elementos técnicos para la toma de decisiones por parte de las autoridades judiciales^(7)^. Esa intervención ocurre de forma interdisciplinaria y, en lo que se refiere a la enfermería, son ejemplos la acogida, la escucha activa, el registro de lesiones físicas, la colecta de rastros biológicos como el semen y la preservación de la integridad de las pruebas^(8)^.

El papel de la enfermería frente a la violencia abarca todos los géneros y grupos de edad; sin embargo, este estudio hace hincapié en la violencia contra las personas mayores (VCPM), considerada un problema de salud pública que requiere intervenciones eficaces. En Brasil, un estudio multicéntrico realizado en dos hospitales con 323 ancianos constató que más de la mitad de los participantes estaban en riesgo de violencia^(9)^.

Por lo tanto, es necesario revelar el potencial y las debilidades del papel de la enfermera en los matices que rodean la gestión de la VCPM, ya que esto muestra la realidad y permite intervenir. De esta forma, preparar a las enfermeras para tratar casos de violencia significa considerar que su trabajo implica una observación atenta de la vida cotidiana de la persona mayor, dándoles la oportunidad de prevenir, detectar y detener la acción^(10)^.

Teniendo en cuenta lo anterior, surgen las siguientes preguntas: ¿cómo intervienen o intervendrán las enfermeras en casos de VCPM en el ámbito hospitalario? ¿Existen diferencias en las intervenciones entre las enfermeras que han atendido y las que no han atendido casos de VCPM? Para responder a estas preguntas, el objetivo fue investigar las intervenciones forenses realizadas por enfermeras con y sin experiencia en la atención a personas mayores en situaciones de violencia. Así, este estudio se justifica por el hecho de que todavía hay poca investigación sobre las intervenciones de las enfermeras en la VCPM en los hospitales, y poner de relieve las debilidades y el potencial de este trabajo es esencial para la toma de decisiones.

## MÉTODO

### Tipo de Estudio

Se trata de un estudio descriptivo transversal con enfoque cuantitativo, basado en las recomendaciones de la herramienta *STrengthening the Reporting of OBservational studies in Epidemiology* (STROBE).

### Lugar del Estudio

El estudio fue realizado en cinco hospitales públicos de João Pessoa, PB, Brasil, seleccionados por atender un número significativo de ancianos, en los sectores de: emergencia, clínica médica, clínica quirúrgica y Unidad de Cuidados Intensivos (UCI).

### Población y Criterios de Selección

La población estaba formada por 660 enfermeros, 285 de ellos distribuidos en los cuatro sectores seleccionados. Los criterios de selección fueron: ser auxiliares de enfermería, con al menos cuatro semanas de experiencia en el sector y trabajar 20 horas semanales. Este período mínimo se consideró necesario para que el profesional estuviera expuesto a situaciones prácticas inherentes a su profesión, lo que le permitiría responder eficazmente a la investigación^(11)^. Se excluyeron las enfermeras que realizaban exclusivamente actividades de gestión y las que estaban de baja por cualquier motivo.

### Definición de la Muestra

Considerando que la población accesible son todas las enfermeras de los cuatro sectores, disponemos de una población finita de tamaño N = 285 personas. Por lo tanto, se consideró una muestra aleatoria simple con una confianza del 98% y un margen de error del 2%. El tamaño de la muestra se obtuvo aplicando la fórmula siguiente, donde z = 1,96; P = 0,50; Q = 1 – P; N = 285 y d = 0,03 y con corrección de población finita mediante la expresión _nf_ ≈ n / [1+ n /N]^(12)^. Así pues, se necesitaban 253 para el muestreo mínimo, pero se recogieron 270.

$$n=\frac{\frac{z^{2}PQ}{d^{2}}}{1+\frac{1}{N}\left(\frac{z^{2}PQ}{d^{2}}-1\right)}$$

### Recogida de Datos

Los datos se recogieron de junio a diciembre de 2022 mediante un cuestionario estructurado elaborado por el investigador y validado por 10 expertos. Para aplicar el instrumento a los participantes, se adoptó un enfoque de conveniencia, reuniéndose con los profesionales en momentos en que estaban disponibles. La investigadora realizó visitas semanales a los departamentos de las instituciones, invitando a participar a las enfermeras en activo. El cuestionario se administró en el lugar indicado por los propios participantes, siguiendo estrictamente el protocolo de uso de mascarilla quirúrgica, de acuerdo con las medidas de prevención del Covid-19.

Para la elaboración del cuestionario se realizó una revisión de alcance «Forensic nursing care for the elderly in situations of violence: scoping review» con el fin de identificar cómo se desarrollan estos cuidados. Los ítems del instrumento mostraron una buena fiabilidad, con un *alfa de Cronbach* superior a 0,70, considerada una buena medida^(13)^, lo que demuestra que el instrumento permite identificar las intervenciones de las enfermeras ante la VCPM. Además, el coeficiente de correlación del punto biserial entre cada ítem presenta valores inferiores a 0,50, mostrando que todos los ítems del instrumento son importantes. La probabilidad de que el participante haya «adivinado» una respuesta, un acierto al azar, fue inferior a 0,04, siendo aceptable hasta 0,05^(14)^.

El cuestionario consistió en datos sociodemográficos y aspectos generales de la VCPM, con 11 ítems, identificados por la letra «A» seguida de un número arábigo del ítem; y acciones realizadas por las enfermeras en casos de VCPM, con 16 ítems, identificados por la letra «B» y una secuencia numérica arábiga con respuestas «sí», «no» y «tal vez». Para la aplicación del instrumento se utilizó un enfoque de conveniencia para conocer a los profesionales, teniendo en cuenta su disponibilidad.

### Análisis y Tratamiento de los Datos

Los datos se organizaron en una hoja de cálculo *Excel* (Microsoft 365, 2019) y se analizaron utilizando el *software* libre Jamovi Versión 0.9, 2020 y SPSS (*Statistical Package for the Social Sciences*) versión 20.0. Se utilizaron frecuencias simples y relativas para caracterizar los datos sociodemográficos y VCPM. Se utilizó la prueba de proporciones para distinguir entre los grupos con y sin experiencia. Si no se podía aplicar esta prueba (proporciones cercanas a 0 o 1), se realizó una comparación mediante la prueba bayesiana de proporciones. Se utilizó la prueba *t-Student* para asociar variables cuantitativas y el análisis estadístico multivariado para diferenciar los ítems con mayor y menor porcentaje de respuestas, utilizando el análisis de correspondencias múltiples (ACM) con los resultados presentados en un mapa perceptual y el análisis de conglomerados utilizando la métrica chi-cuadrado, con agrupamiento jerárquico y el método de enlace promedio en un dendograma^(14,15)^. La consistencia interna del instrumento se analizó mediante *el alfa de Cronbach* con un intervalo de confianza del 95%.

### Aspectos Éticos

Esta investigación forma parte del proyecto Instrumentalización de la enfermería forense en el cuidado de ancianos hospitalizados, aprobado por el Comité de Ética de la Investigación bajo el dictamen nº 3.709.600 y CAAE nº 10179719.9.0000.5183, de acuerdo con la Resolución 466/2012 del Consejo Nacional de Salud.

## RESULTADOS

Participaron de la investigación 270 enfermeros. De ellos, 203 (75%) no tenían experiencia con VCPM y 67 (25%) sí. En ambos grupos -con y sin experiencia en VCPM- la mayoría de los participantes eran mujeres (236 – 87,4%), con edades comprendidas entre 31 y 40 años (172 – 63,8%), especialistas (244 – 90,4%), que trabajaban en el sector de urgencias (103 – 38,1%) y en medicina clínica (69 – 25,6%), con al menos dos puestos de trabajo (204 – 75,5%). En cuanto a la antigüedad, los participantes sin experiencia en VCPM habían trabajado en el sector durante más de diez años (60 – 29,5%), mientras que 30 (44,7%) de los que tenían experiencia en VCPI habían trabajado en el sector entre tres y seis años, según la Tabla 1.

**Tabla 1:** Variables sociodemográficas de los enfermeros – João Pessoa, PB, Brasil, 2022.

Variable	Categoría	Sin		Con		Total	
		N	%	N	%	N	%
**Sexo**	Masculino	24	8,8	10	3,7	34	12,6
	Femenino	179	66,2	57	21,1	236	87,4
**Edad**	18 a 30	4	1,5	14	5,1	18	6,7
	31 a 40	131	48,5	41	15,1	172	63,8
	41 a 50	67	24,8	8	2,9	75	27,8
	60 a 70	1	0,4	4	1,5	5	1,9
**Escolaridad**	Doctorado	2	0,7	1	0,3	3	1,1
	Maestría	5	1,8	4	1,5	9	3,3
	Especialista	188	69,6	56	20,7	244	90,4
	Graduado	8	2,9	6	2,2	14	5,2
**Sector**	Clínica Quirúrgica	38	14,0	10	3,7	48	17,8
	Clínica Médica	40	14,8	29	10,7	69	25,6
	Urgencia	89	32,9	14	5,1	103	38,1
	CTI	36	13,3	14	5,1	50	18,5
**Cantidad de puestos**	Uno	7	2,6	21	7,8	28	10,4
	Dos	165	61,1	39	14,4	204	75,5
	Tres	30	11,1	7	2,59	37	13,7
	Cuatro	1	0,3	0	0,0	1	0,3
**Tiempo en el sector (en años)**	=1	10	4,9	5	7,4	15	5,6
	1 a 3	55	27,0	15	22,3	70	25,9
	3 a 6	38	18,7	30	44,7	68	25,2
	6 a 9	40	19,7	7	10,4	47	17,4
	=10	60	29,5	10	14,9	70	25,9

En cuanto a los aspectos generales de la VCPM, sólo el ítem A5 mostró respuestas similares entre los dos grupos, y una diferencia significativa de p < 0,005 en los demás ítems, como se muestra en la Tabla 2.

**Tabla 2:** Aspectos generales de la VCPM considerando las respuestas de los dos grupos – João Pessoa, PB, Brasil, 2022.

Ítem	Categoría	Sin		Con		Total		Valor-P test de Fisher
		N	%	N	%	N	%	
A1 – ¿Ha detectado algún caso o sospecha de violencia contra la persona mayor?	**Si**	0	0,0	67	100	67	24,8	
	**No**	198	97,5	0	0,0	198	73,3	< 0,001*
	**Quizás**	5	2,5	0	0,0	5	1,9	
A2 – ¿Ha notado malestar por parte de la persona mayor en presencia de sus familiares?	**Si**	74	36,5	62	92,5	136	50,4	
	**No**	128	63,1	4	6,0	132	48,9	< 0,001*
	**Quizás**	1	0,5	1	1,5	2	0,7	
A3 – ¿Le resulta fácil detectar que la persona mayor se encuentra en una situación de violencia?	**Si**	35	17,2	19	28,4	54	20,0	
	**No**	165	81,3	42	62,7	207	76,7	0,0014*
	**Quizás**	3	1,5	6	9,0	9	3,3	
A4 – ¿Conoce alguna herramienta o escala que ayude en la atención a personas mayores con sospecha o confirmación de violencia? (aunque nunca las haya utilizado)	**Si**	2	1,0	9	13,4	11	4,1	
	**No**	201	99,0	56	83,6	257	95,2	
	**Quizás**	0	0,0	2	3,0	2	0,7	< 0,001*
A5 – ¿Considera importante disponer de algún material (protocolo, escala, instrumento) para ayudar a atender a las personas mayores víctimas de violencia?	**Si**	201	99,0	66	68,5	267	98,9	
	**No**	2	1,0	1	1,5	3	1,1	0,9999*
	**Quizás**	0	0,0	0	0,0	0	0,0	
A6 – ¿Utiliza algún instrumento o escala que ayude en la atención a personas mayores con sospecha o confirmación de violencia?	**Si**	1	0,8	5	7,5	6	2,2	
	**No**	201	99,5	60	89,5	262	97,0	
	**Quizás**	0	0,0	2	3,0	2	0,7	0,0004*
A7 – ¿Se siente preparada/o para identificar situaciones de violencia contra las personas mayores?	**Si**	63	31	11	16,4	74	27,4	
	**No**	133	65,5	47	70,1	180	66,7	0,0026*
	**Quizás**	7	3,5	9	13,4	16	5,9	
A8 – ¿Cree que hay suficiente formación para que los enfermeros identifiquen situaciones de violencia contra los ancianos?	**Si**	0	0,0	7	10,4	7	2,6	
	**No**	203	100	59	88,1	262	97,0	
	**Quizás**	0	0,0	1	1,5	1	0,4	< 0,001*
A9 – ¿Cree que las personas mayores ocultan que han sufrido violencia?	**Si**	146	71,9	62	92,5	234	86,7	
	**No**	57	77,3	3	4,5	33	12,3	< 0,001*
	**Quizás**	0	0,0	2	3,0	3	1,1	
A10 – ¿Ha presenciado alguna vez situaciones en las que la persona mayor dice estar mejor atendida en el hospital que en su entorno familiar?	**Si**	146	71,9	62	92,5	108	40	
	**No**	57	28,0	3	4,5	160	59,3	
	**Quizás**	0	0,0	2	3,0	2	0,7	< 0,001*
A11 – ¿Cree que la violencia perjudica la mejoría de la persona mayor cuando está hospitalizada?	**Si**	172	84,7	62	92,5	234	86,7	
	**No**	31	15,3	3	4,5	3	1,1	
	**Quizás**	0	0,0	2	3,0	33	12,2	0,0033*

*Prueba de Fisher. A = categorización de los ítems.

La tabla 3 muestra que existe una diferencia significativa en la mayoría de los ítems de ambos grupos, excepto en los ítems B1 y B5. Al final de la tabla 3, la *prueba t de Student,* adecuada para muestras grandes cuando se comparan medias, confirma que existe una diferencia significativa entre las puntuaciones obtenidas por los dos grupos.

**Tabla 3:** Comparación de las intervenciones de enfermeros con y sin experiencia en VCPM – João Pessoa, PB, Brasil, 2022.

Ítem	Categoría	Sin		Con		Total		Valor-P Chi-cuadrado
		N	%	N	%	N	%	
B1 – ¿Ha remitido o remitiría los casos sospechosos o confirmados a Asistente social?	**Si** **No** **Quizás**	203 0 0	100 0 0	65 2 0	97,0 2,9 0	268 2 0	98,9 0,7 0,0	0,3075
B2 – ¿Remitió o remitiría los casos sospechosos o confirmados a la policía?	**Si** **No** **Quizás**	11 189 3	5,4 93,1 1,5	26 38 3	38,8 56,7 4,4	37 225 8	13,7 83,6 2,9	< 0,001*
B3 – ¿Ha remitido o remitiría casos sospechosos o confirmados al Consejo de Mayores?	**Si** **No** **Quizás**	13 189 1	6,4 93,1 0,5	26 40 4	38,8 59,7 6	39 224 7	14,4 83,3 2,6	< 0,001*
B4 – ¿Realiza una anamnesis/entrevista con la persona mayor para evaluar los signos y síntomas de violencia?	**Si** **No** **Quizás**	201 1 0	99,0 0,5 0,0	59 7 1	88,1 10,4 1,5	260 7 3	95,9 2,6 2,1	< 0,001*
B5 – ¿Realiza escuchas cualificadas para tratar de identificar presuntos casos de violencia contra las personas mayores?	**Si** **No** **Quizás**	171 30 1	83,3 14,7 0,5	60 4 3	89,6 5,9 4,4	229 35 6	84,8 12,9 2,2	0,3429
B6 – ¿Realiza un examen físico de los mayores para evaluar signos y síntomas de violencia?	**Si** **No** **Quizás**	112 46 30	54,9 22,6 14,7	61 4 2	91,0 5,9 3	173 50 32	64,0 18,5 11,8	< 0,001*
B7 – ¿Registra o registraría los casos sospechosos o confirmados en los historiales médicos?	**Si** **No** **Quizás**	201 2 0	98,5 0,9 0,0	58 4 5	86,6 5,9 7,4	259 5 6	95,9 1,8 2,2	< 0,001*
B8 – ¿Elabora o elaboraría planes de atención para personas mayores y familias implicadas en situaciones de malos tratos, abusos sexuales, traumas y otras formas de violencia?	**Si** **No** **Quizás**	42 160 1	20,6 78,8 0,5	27 39 1	40,3 58,2 1,5	69 199 2	25,5 73,7 0,7	0,0023*
B9 – ¿Acogen a ancianos víctimas de violencia sexual, traumas y otras formas de violencia?	**Si** **No** **Quizás**	203 0 0	100 0,0 0,0	63 3 1	94,0 4,5 1,5	266 4 0	98,5 1,5 0,0	0,0178*
B10 – ¿Interroga a la familia del anciano en caso de sospecha o confirmación?	**Si** **No** **Quizás**	103 52 40	50,7 25,6 19,7	57 5 5	85,1 7,4 7,4	160 57 45	59,2 21,1 16,6	< 0,001*
B11 – ¿Informa al equipo multiprofesional de los casos de violencia presunta o confirmada contra las personas mayores para intentar resolver el problema?	**Si** **No** **Quizás**	203 0 0	100 0,0 0,0	61 5 1	91,0 7,4 1,5	265 4 1	98,1 1,4 0,3	0,667B**
B12 – En caso de sospecha o confirmación de violencia contra las personas mayores, ¿informa al médico de la situación para que le ayude a tratarla?	**Si** **No** **Quizás**	203 0 0	100 0,0 0,0	62 4 1	92,5 1,4 1,5	265 4 1	98,1 1,4 0,3	0,130B**
B13 – Colabora con la justicia en los casos necesarios.	**Si** **No** **Quizás**	171 30 2	83,8 41,2 0,9	59 5 3	88,1 7,4 4,4	230 34 6	85,1 12,6 2,2	0,0039*
B14 – Reconoce posibles situaciones de violencia contra las personas mayores, identifica víctimas potenciales y realiza diagnósticos enfermeros en el contexto de abusos, traumas, violencia sexual y otras formas de violencia.	**Si** **No** **Quizás**	10 192 1	4,9 94,5 0,5	36 27 4	53,7 40 6	46 216 8	17,0 80 2,9	< 0,001*
B15 – Promueve o promovería la protección de los derechos humanos y las garantías legales de los ancianos, sus familias y las personas que cometieron la violencia.	**Si** **No** **Quizás**	196 2 5	96,1 0,9 2,4	59 3 5	88,1 4,5 7,4	255 6 9	94,4 2,2 3,3	0,0340*
B16 – Preserva o preservaría rastros en casos de malos tratos, violencia sexual y otras formas de violencia con el fin de probar la violencia contra las personas mayores.	**Si** **No** **Quizás**	13 187 3	6,4 92,1 1,4	27 38 2	40,2 56,7 2,9	54 208 8	20 77,0 2,9	< 0,001*
**Media**		10,0		12,2		10,6		**Valor-P**
**Mediana**		11,0		12,0		11,0		0,0020*t
**Desvío Estándar**		1,7		3,0		2,3		

*Diferencia significativa al nivel del 5% - prueba de Chi-cuadrado.

**B = Prueba bayesiana de dos proporciones utilizada.

*t = prueba t de Student. B = categorización de ítems.

Se puede observar que existen ítems con altos valores de porcentaje de respuesta y otros con valores muy bajos en la Tabla 3,

y para decidir qué ítems se diferencian con alguna de estas caracterizaciones, se utilizó el análisis estadístico multivariante, aplicando los modelos ACM^(15)^ y análisis de conglomerados ^(16)^.

Los resultados del ACM presentados en la Tabla 4 permiten concluir que una única dimensión de ítems de la Tabla 3 explica el 98,4% de la variabilidad total contenida en la información de la Tabla 3. Por lo tanto, la dimensión 2 (ítems: B1, B4, B5, B7, B9, B11, B12, B15) tiene ítems más asociados a respuestas afirmativas.

**Tabla 4:** Análisis de Correspondencias Múltiples de las respuestas de la Tabla 3 – João Pessoa, PB, Brasil, 2022.

Dimensión	Valor singular	Inercia	Chi-cuadrado*	Valor-P	% Inercia
1	0,096	0,009	2379,93	< 0,001	1,6
2	0,740	0,548			98,4
Total		0,557			100,0

La Figura 1 muestra el mapa perceptual en dos dimensiones, donde se puede observar que existen tres clusters cuyo significado, en el MCA, es que cuanto mayor es la proximidad entre los niveles de las categorías de la Tabla 3, mayor es la asociación entre estos elementos que forman el cluster^(16)^. El cluster más grande está formado por la asociación entre los elementos B1, B4, B5, B7, B9, B11, B12, B13 y B15 y la respuesta «sí».

**Figura 1:** Mapa perceptual del ACM y dendrograma del análisis de conglomerados de la Tabla 3 – João Pessoa, PB, Brasil, 2022.

Se observa que la característica común a todos estos ítems es su alto porcentaje de aparición. Los ítems B6 y B10 están más asociados a respuestas «tal vez» para el grupo sin experiencia en VCPM y, por último, el cluster de la derecha, formado por los ítems B2, B3, B8, B14 y B16, es el que tiene menor porcentaje de respuestas «sí» y está más asociado a respuestas «no» para ambos grupos.

El análisis cluster utilizando la métrica Chi-Cuadrado con clustering jerárquico y el método average linkage^(15)^ tiene su dendrograma mostrado junto al mapa perceptual, y hay un corte vertical a una distancia igual a 10, donde se pueden ver los mismos grupos de ítems mostrados en el MCA, confirmando la misma interpretación dada a los resultados mostrados en el mapa perceptual.

## DISCUSIÓN

Se reveló que, en relación a los aspectos generales de la VCPM, la mayoría de las enfermeras de ambos grupos ya había notado malestar en el anciano en presencia de un familiar, encontró difícil detectar la VCPM, relató no conocer y no utilizar instrumentos y/o protocolos para ayudar a identificar e intervenir en los casos y consideró importante tenerlos. Además, las enfermeras no se sienten preparadas para identificar la VCPM, señalan que no hay suficiente oferta formativa para prepararlas, lo que dificulta el abordaje de los casos, ya que la persona mayor oculta la violencia.

En línea con estas conclusiones, los estudios sobre las percepciones de los profesionales de enfermería en relación con la detección y prevención de la VCPM revelan que, aunque muchos consideran o sospechan la existencia de casos, tienen dificultades para actuar, especialmente debido a la falta de formación específica y a la ausencia de integración entre los servicios públicos destinados a este tipo de atención. También expresan su preocupación por la falta de formación^(17,18)^.

Frente a la falta de preparación revelada en este estudio y en otras investigaciones, es importante destacar que las enfermeras desempeñan un papel importante en las implicaciones legales de los casos forenses y, para ello, necesitan adquirir conocimientos de enfermería en ciencias forenses^(19)^. Además, el uso de protocolos e instrumentos que guíen la realización del VCPM son fundamentales para una atención adecuada^(20)^.

En este estudio, se observó que la mayoría de las enfermeras revelaron que habían sido testigos de cómo los ancianos informaban de que estaban mejor atendidos en el entorno hospitalario que en sus propios hogares. Esto puede revelar que el 71% de los profesionales que declararon no haber identificado nunca una situación de VCPM podrían, de hecho, haberse enfrentado ya a un caso de este tipo de violencia, pero no supieron identificarlo o no lo investigaron. Corroborando estas conclusiones, otro estudio señala que identificar los casos no es fácil. Por eso es importante que las enfermeras conozcan los principios de la ciencia forense para poder prestar la asistencia adecuada cuando se encuentren con una víctima^(21)^.

Las lagunas en la gestión de estos casos suelen ser el resultado de una combinación de factores, como el desconocimiento de los protocolos y la falta de formación especializada. Destacan las dificultades a las que se enfrentan los profesionales tanto en el reconocimiento precoz de la violencia como en el enlace con las redes de apoyo y protección, lo que pone de manifiesto la necesidad de normalización y de aplicación de protocolos claros para el tratamiento de la VCPM^(22)^.

Las prácticas asistenciales reveladas en este estudio muestran un distanciamiento del ideal preconizado por las políticas públicas en relación a la gestión de la violencia. Esto resalta la necesidad urgente de invertir en la capacitación de los profesionales y en la implementación de flujogramas que orienten la atención en los diferentes sectores. Esta iniciativa debe complementarse con la ampliación de los espacios de coordinación intersectorial y la creación de un sistema de comunicación institucional eficiente entre los diversos actores involucrados. Este esfuerzo debe superar el desajuste entre las ideas concebidas, las acciones realizadas y los lineamientos establecidos^(23)^. Además de lo anterior, se constató que la mayoría de las enfermeras de ambos grupos remiten o remitirían los casos de VCPM al servicio social y a otros miembros del equipo multiprofesional. Esto es importante para que cada profesional pueda actuar dentro de sus competencias para resolver la situación. Sin embargo, cuando se trata de remitir el caso a la policía y al Consejo de Mayores, la mayoría en ambos grupos no lo hace o no lo haría, lo que va en contra de la recomendación ético-legal, que establece que los casos sospechosos o confirmados de VCPM deben ser comunicados a estos organismos^(24)^.

En cuanto a la realización de anamnesis para valorar signos de violencia, casi todas las enfermeras realizan o realizarían estas acciones, así como la realización de escucha y acogida cualificadas. Estas prácticas, si son realizadas con calidad, permiten identificar la violencia y subsidiar la planificación de los cuidados, pues según la literatura, las respuestas verbales y no verbales también son evidencias y, por lo tanto, deben ser documentadas en los registros de enfermería^(25)^.

El registro de VCPM está presente en la mayoría de las respuestas, pero la mayoría no elabora o no elaboraría planes de cuidados para las víctimas, diagnósticos de enfermería o no reconocería víctimas potenciales. Además, casi la mitad de los enfermeros sin experiencia no interrogarían a la familia de la víctima, lo que revela importantes lagunas en la gestión eficaz de los casos. Los profesionales sanitarios que trabajan en unidades hospitalarias, especialmente en urgencias y UCI, tienen más probabilidades de encontrarse con situaciones forenses, lo que requiere que sean capaces de recopilar datos relevantes para orientar la planificación de los cuidados^(26)^, ya que esto forma parte del trabajo de la enfermera.

En ambos grupos se realizó una exploración física. La anamnesis, la escucha, la acogida y el examen físico son esenciales en la profesión para poder llevar a cabo el proceso de enfermería, y deberían formar parte de la rutina de toda enfermera. Cuando se trata de sospechas de violencia, se recomienda que las enfermeras realicen entrevistas de enfermería forense y recojan pruebas mediante una evaluación de pies a cabeza^(19)^. La eficacia de esta evaluación es importante, porque además de los aspectos verbales y no verbales, el aspecto y la localización de las lesiones pueden contar la historia del trauma físico, incluyendo si es agudo o crónico, así como otros malos tratos. La obtención de estos detalles mediante un examen físico, registros e imágenes apropiadas puede aportar pruebas que corroboren o refuten una sospecha de violencia. Por lo tanto, la calidad de esta evaluación tiene un impacto directo en las investigaciones^(27)^.

Además, la mayoría de los participantes revelaron que colaboran o colaborarían con la justicia para resolver los casos; sin embargo, cuando se les preguntó si conservan o conservarían rastros, hubo una baja respuesta afirmativa, lo que corrobora un estudio realizado en Nueva Zelanda, que reveló una limitación en los conocimientos de los enfermeros para identificar correctamente los casos forenses, especialmente en lo que respecta a los aspectos ético-legales inherentes a la atención clínica, la recogida y la conservación de rastros^(19)^. Esto pone de relieve la necesidad de que las enfermeras preserven y recojan pruebas durante la prestación de los cuidados para que se puedan llevar a cabo los procedimientos judiciales^(19)^ ya que, como reveló otro estudio, las posibilidades de que la víctima denuncie la violencia aumentan 2,5 veces cuando la lesión está documentada^(28)^.

En cuanto a las intervenciones en los casos de VCPM destacados en esta investigación, se constató que existen lagunas en la gestión. Las intervenciones forenses realizadas por enfermeros, esenciales en el manejo de la violencia contra personas mayores, presentan debilidades, especialmente cuando se trata de la recolección de pruebas y de la planificación de cuidados que garanticen la seguridad y el respeto a la víctima. La formación insuficiente y la ausencia de protocolos claros ponen en peligro el manejo adecuado de los casos ^(5,19)^. Prácticas como la escucha activa, el apoyo emocional y la integración con redes de apoyo, aunque relevantes, están todavía infrautilizadas^(1,17)^.

En este estudio se constató que el grupo con experiencia en VCPM llevó a cabo acciones específicas de Enfermería Forense. Las intervenciones incluyeron la colaboración con el sistema judicial, promoviendo la protección de los derechos humanos y las garantías jurídicas de las víctimas, sus familiares y las personas que cometieron la violencia, así como la preservación de rastros en casos de malos tratos, violencia sexual y otras formas de violencia para probar la violencia contra las personas mayores. Estas prácticas refuerzan la importancia de las enfermeras forenses como agentes que integran los cuidados, la justicia y la ciencia, promoviendo una asistencia humanizada y contribuyendo a la construcción de un sistema sanitario más justo y eficaz^(21)^.

Una de las limitaciones de este estudio fue la dificultad de las enfermeras en reconocer la VCPM, pues se referían a situaciones características de violencia, sin comprender que verbalizar tales atributos caracteriza la sospecha del fenómeno. Además, el estudio fue realizado en un ambiente hospitalario, por lo tanto, puede no representar la realidad de la Atención Primaria. Partiendo de este entendimiento, se cree que la divulgación de los resultados de esta investigación posibilitará avances científicos en salud y enfermería, ayudando así a orientar a los gestores en el desarrollo de entrenamientos dirigidos a ampliar las competencias de las enfermeras que atienden ancianos en situación de violencia, y así mejorar los cuidados prestados.

## CONCLUSIÓN

Los resultados de este estudio muestran cómo las enfermeras hospitalarias con y sin experiencia en VCPM intervienen o intervendrán, respectivamente, en estos casos de violencia. Al comparar los dos grupos, se observó una similitud significativa entre ellos, con la mayoría de las enfermeras con experiencia realizando intervenciones importantes en la gestión de los casos, a pesar de llevar a cabo acciones esenciales con debilidades, como la realización de derivaciones a las autoridades, así como la notificación a organizaciones de apoyo como el Consejo de Mayores.

Esto pone de manifiesto la necesidad de utilizar herramientas y proporcionar formación para orientar la atención de las enfermeras a los ancianos. Por lo tanto, se sugiere que se realicen investigaciones e intervenciones para mejorar el manejo de las enfermeras en: la remisión del caso a la policía; la elaboración de un plan de cuidados y diagnósticos de enfermería; el interrogatorio a la familia del anciano; el reconocimiento de situaciones de violencia y la preservación de rastros, que son acciones esenciales para interrumpir el ciclo de violencia, pero que se manejan con debilidad. También es necesario dar mayor difusión a la especialización en EF.
